# Transcriptomic Profiling Reveals Shared Signalling Networks Between Flower Development and Herbivory-Induced Responses in Tomato

**DOI:** 10.3389/fpls.2021.722810

**Published:** 2021-09-21

**Authors:** Lanlan Ke, Yangzi Wang, Martin Schäfer, Thomas Städler, Rensen Zeng, Jörg Fabian, Hannier Pulido, Consuelo M. De Moraes, Yuanyuan Song, Shuqing Xu

**Affiliations:** ^1^Key Laboratory of Ministry of Education for Genetics, Breeding and Multiple Utilization of Crops, College of Life Sciences, Fujian Agriculture and Forestry University, Fuzhou, China; ^2^Institute for Evolution and Biodiversity, University of Münster, Münster, Germany; ^3^Plant Ecological Genetics Group, Institute of Integrative Biology, ETH Zürich, Zürich, Switzerland; ^4^Institute for Pharmaceutical and Medicinal Chemistry, University of Münster, Münster, Germany; ^5^Department of Environmental Systems Sciences, ETH Zürich, Zürich, Switzerland

**Keywords:** herbivory, defence response, flower development, pollinator attraction, pleiotropy, jasmonic acid, signalling networks, *Solanum lycopersicum*

## Abstract

Most flowering plants must defend themselves against herbivores for survival and attract pollinators for reproduction. Although traits involved in plant defence and pollinator attraction are often localised in leaves and flowers, respectively, they will show a diffuse evolution if they share the same molecular machinery and regulatory networks. We performed RNA-sequencing to characterise and compare transcriptomic changes involved in herbivory-induced defences and flower development, in tomato leaves and flowers, respectively. We found that both the herbivory-induced responses and flower development involved alterations in jasmonic acid signalling, suppression of primary metabolism and reprogramming of secondary metabolism. We identified 411 genes that were involved in both processes, a number significantly higher than expected by chance. Genetic manipulation of key regulators of induced defences also led to the expression changes in the same genes in both leaves and flowers. Targeted metabolomic analysis showed that among closely related tomato species, jasmonic acid and α-tomatine are correlated in flower buds and herbivory-induced leaves. These findings suggest that herbivory-induced responses and flower development share a common molecular machinery and likely have coevolved in nature.

## Introduction

Most plants have to protect themselves from insect herbivory for survival and attract pollinators for reproduction. While the defence against insect herbivores is often mediated by toxic chemical compounds and physical barriers in leaves (Wu and Baldwin, 2010), pollinator attraction is mostly achieved by various floral signals, such as colours and scents produced by flowers (Fenster et al., 2004; Sheehan et al., 2012; Fattorini and Glover, 2020). The spatial separation of tissues that mediate plant defence and pollinator attraction resulted in often isolated studies on the molecular mechanisms and evolution of traits involved in defence and floral signals, but see examples in Strauss and Irwin (2004); Pashalidou et al. (2020). However, if plant defence and floral signals are regulated by the same molecular machinery, they will show diffuse coevolution due to pleiotropy (Iwao and Rausher, 1997; Strauss and Irwin, 2004; Agrawal and Stinchcombe, 2009; Wise and Rausher, 2013). For example, in *Cardamine hirsuta*, allelic variations at the major floral repressor *Flowering Locus C* affect both flowering time and plant defences, such as total glucosinolates and stress-related phytohormones in leaves (Rasmann et al., 2018). Therefore, in a hypothetic population, the flowering time of *C. hirsuta* will evolve under joint selection imposed by both pollinators and herbivores. The same is also true for defences. Indeed, recent experimental evolution studies in *Brassica rapa* found that floral signals and plant defence evolved *via* diffuse coevolution, likely due to pleiotropic effects (Zu et al., 2016; Ramos and Schiestl, 2019). For example, plants under selection by bee pollinators evolved increased floral attractiveness, but the process was compromised by the presence of herbivores. However, the underlying molecular mechanisms remain unclear.

As plant defence often involves costs, many plants evolved induced defence strategies that are activated only upon attack from herbivores (Simms and Fritz, 1990; Wu and Baldwin, 2010; Fürstenberg-Hägg et al., 2013). Herbivory-induced responses are often mediated by jasmonic acid (JA), a fatty acid-derived plant hormone crucial for regulating the plant defence against various herbivores (Walling, 2000; Gatehouse, 2002; War et al., 2012; Fürstenberg-Hägg et al., 2013; Wang and Wu, 2013; Zhang et al., 2017; Aljbory and Chen, 2018; Erb and Reymond, 2019; Wang et al., 2019). The rapid accumulation of JA and its derivatives, such as jasmonyl-isoleucine (JA-Ile) and methyl-jasmonate (MeJA), is a conserved response in herbivore-damaged plants (Al-Zahrani et al., 2020; Zhang et al., 2020). In turn, mutants deficient in JA biosynthesis, perception or signal transduction exhibit increased susceptibility to herbivores, shown, e.g. in wild tobacco (Kessler et al., 2004), tomato (Li et al., 2005; Bosch et al., 2014) and *Arabidopsis* (McConn et al., 1997; Chung et al., 2008). The elicited JA signalling can activate a cascade of metabolic reprogramming that involves the biosynthesis of diverse bioactive specialised compounds (Howe, 2004; Memelink, 2009; De Geyter et al., 2012; Wasternack and Hause, 2013; Durrant et al., 2017), such as phenolics, alkaloids and glucosinolates, and defence proteins, such as proteinase inhibitors (PI), threonine deaminase (TD), cathepsin D inhibitor (CDI), arginase (ARG), leucyl aminopeptidase (LAP) and polyphenol oxidase (PPO), which are toxic, repellent or anti-nutritional to herbivores (Bennett and Wallsgrove, 1994; Chen et al., 2005; Fowler et al., 2009; Chung and Felton, 2011; War et al., 2012; Fürstenberg-Hägg et al., 2013; Paudel et al., 2019). In addition, herbivore attacks can also activate the emission of volatile organic compounds, such as terpenoids and green-leaf volatiles that act as indirect defences (De Moraes et al., 2001; Schnee et al., 2006; War et al., 2012; Fürstenberg-Hägg et al., 2013; Aljbory and Chen, 2018).

Interestingly, similar molecular and metabolic processes are involved in herbivory-induced defence responses, as well as during flower development and opening. JA signalling is required for flower development (Wasternack et al., 2013; Yuan and Zhang, 2015; Huang et al., 2017). For example, in *Arabidopsis thaliana*, the JA-deficient *dad1* mutant shows slower flower development, delayed anther dehiscence and reduced viability of pollen grains (Ishiguro et al., 2001). In wild tobacco, the floral limbs cannot fully open when JA biosynthesis is interrupted (Stitz et al., 2014). A similar pattern is also found in Chinese cabbage (*Brassica campestris*; Peng et al., 2019) and rice (Xiao et al., 2014). Recently, a study in tomato suggests that the JA-mediated flower development and opening are mediated by *SlMYB21* (Niwa et al., 2018). Flower development and opening also involve serious changes in the primary and secondary metabolisms. During flower development, the chromoplasts gradually increase (Giuliano et al., 1993) resulting in a progressively elevated biosynthesis of carotenoids and other coloured substances to produce colourful flowers. In addition, flower opening coincides with petal expansion, which involves hydrolysis of stored carbohydrates (Bieleski, 1993; Bieleski et al., 2000; Vergauwen et al., 2000; Yap et al., 2008), and release of volatile organic compounds from petals and nectar (González-teuber and Heil, 2009; Heil, 2011; Dudareva et al., 2013).

Recent studies have shown that herbivory-induced defence responses and flower development not only involve a similar phytohormonal regulation and metabolic rearrangement, but also that they can even share the same molecular machinery. For instance, *CYP82G1*, which is expressed in both herbivory-induced leaves and flowers, is required for the biosynthesis of a homoterpene emitted from both respective tissues in *Arabidopsis* (Lee et al., 2010). Similarly, in wild tobacco, tissue-specific expression of *NaTPS38* is required for the emission of (*E*)-α-bergamotene from leaves after herbivory and from flowers (Zhou et al., 2017; Xu et al., 2020). Likewise, changes in diterpene glycoside biosynthesis alter both the anti-herbivore defence and flower development in the wild tobacco (Li et al., 2021). In tomato and potato, altering the biosynthesis of steroidal glycoalkaloids (SGA), one of the major chemical defences in *Solanum* suppresses flower development (Itkin et al., 2011; Umemoto et al., 2016). However, it remains unclear to what extent herbivory-induced responses and flower development share the same molecular machinery at the genome-wide level.

Here, we aim to address this challenge using comparative transcriptomic profiling. To this end, we sequenced the transcriptomes of control and tobacco hornworm treated leaves, as well as flower tissues at two different developmental stages in three genotypes of domesticated tomato (WT, JA biosynthesis mutant *spr8* and the transgenic *Prosystemin* (*PS*) over-expression line *35S::PS*). The results revealed that a large number of genes, higher than expected by chance, was shared between herbivory-induced responses and flower development. In addition, targeted metabolomic analysis showed correlated changes of JA or α-tomatine in flower buds and herbivory-induced leaves among six closely related species, suggesting diffuse coevolution due to gene pleiotropy in tomatoes.

## Materials and Methods

### Plant Materials and RNA Isolation

Tomato (*Solanum lycopersicum* L.) cv. Castlemart (CM) was used as the wild type (WT). The mutant plants *35S::PS* and *spr8* used in this study were previously described (Yan et al., 2013). Six wild tomato species used in this study included *S. arcanum*, *S. chilense*, *S. chmielewskii*, *S. corneliomulleri*, *S. habrochaites* and *S. neorickii*.

Plants were grown from seeds in an insect-free glasshouse at ETHZ (Lindau-Eschikon, canton Zurich, Switzerland). They were potted in 1-L pots using fresh soil (Ricoter Substrate 214, Ricoter Erdaufbereitung AG, Aarberg, Switzerland)^1^ and fertiliser granules (Gartensegen, Hauert HBG Dünger AG, Grossaffoltern, Switzerland).^2^ Plants were watered two–four times per week. All plants were grown in a greenhouse with a temperature of 20°C at daytime and 16°C at night, with 12h light at 18 kLux and 50% relative humidity.

The leaf samples were collected 6weeks after germination. Herbivory treatments were performed using *Manduca sexta*, which is a specialist herbivore of Solanaceae. *Manduca sexta* eggs were obtained from an in-house colony of the Max Planck Institute for Chemical Ecology, Jena, Germany. One *M. sexta* neonate was placed on each of four selected leaves per plant. After ~24h, three well-damaged leaves were selected per plant, the damage level on each of those leaves was recorded and they were separately collected and immediately frozen in liquid nitrogen. On average, ~24h herbivory resulted in ~10% leaf damage. Comparable leaves from untreated plants were used as control. Three replicates (plants) were analysed for each genotype and treatment.

Flower sampling was conducted 11–12weeks after the herbivory sampling (when most of the plants started flowering). To synchronise flowering time, we transferred all plants to 3-L pots and shifted to a fully controlled climate chamber with a temperature of 22°C at daytime and 18°C at night, with 10h light 4–5weeks before sampling. To avoid the confounding effects of herbivory on flower development, we only collected flower samples from the control plants (plants that were not treated with herbivory). Different flower stages of the same plants were collected on the same day (all between 13:00–16:00) and frozen in liquid nitrogen. The two developmental stages were identified according to a previous study (Dobritzsch et al., 2015). The fully opened flowers were considered as stage 5, whereas the flower bud located next the fully opened flowers on the same inflorescence was considered as stage 4.

Three replicates were collected for each genotype per developmental stage. In total, 18 flower samples (3 genotypes * 3 replicates * 2 developmental stages) were collected. Both leaf and flower samples were stored in a −80°C freezer until RNA extraction.

Total RNA was isolated using the NucleoSpin RNA Plant kit (Macherey-Nagel) and subsequently treated with RNase-free DNase to remove all genomic DNA contamination. RNA quality was analysed *via* NanoDrop (OD260/280 and OD260/230) and Agilent 2100 Bioanalyzer (concentration, 28S/18S, RIN) by Beijing Genomics Institute (BGI, Shenzhen, China).

### RNA-Sequencing

In total, 36 cDNA libraries (18 from leaves: 3 genotypes * 3 replicates * 2 treatments; 18 from flowers: 3 genotypes * 3 replicates * 2 developmental stages) were generated and sequenced on the Illumina HiSeq X Ten platform at the BGI-Shenzhen. On average, 42.6 million paired-end clean reads (150bp) per library were obtained after filtering the low-quality reads from the raw data. Then, TopHat2 (Kim et al., 2013) with the following parameters *--segment-length 30 --segment-mismatches 3 --read-gap-length 5 -m 1 -N 10 --read-edit-dist 7 -r 50 --mate-std-dev 50* was used to map the sequences to the reference genome which was downloaded from ftp://ftp.solgenomics.net/genomes/Solanum_lycopersicum/assembly/build_3.00/. The mapping rate ranged from 90.5 to 95.2% of each library (on average, 93.2%). Following mapping, StringTie (v1.0.4; Pertea et al., 2015) was used to reconstruct and assemble the transcripts. The expression abundance of each gene was calculated using RSEM (v1.2.12; Li and Dewey, 2011) and presented as transcripts per million reads (TPM). The raw sequencing data of 36 samples were submitted to the National Center for Biotechnology Information (NCBI) Sequence Read Archive (SRA)^3^ under accession number PRJNA600385.

### Differential Gene Expression Analysis

The R package edgeR (v3.26.8; Robinson et al., 2010) was used to identify differentially expressed genes (DEGs). Raw count data for each pair of comparison groups were used as input. Genes with counts greater than 10 in at least three samples were considered in the downstream analysis, while all others were defined as lowly expressed genes and removed. The criterion for being considered a DEG was a gene with an absolute fold change of more than two (|log_2_FoldChange|≥1) and a false discovery rate (FDR)≤0.05.

Herbivory-responsive genes were defined as genes that show differential expression between control samples and herbivory samples (control vs. herbivory) in WT plants. Flowering-associated genes were defined as genes that show differential expression between the two developmental stages in WT plants.

Heat maps were drawn by the R packages pheatmap (v1.0.12; Kolde, 2019) and TBtools (v1.075; Chen et al., 2020), with Log_10_(TPM+1) as input. The expression values were scaled across samples for each gene. Venn diagrams were generated with the R package VennDiagram (v1.6.20; Chen, 2018).

### Permutation Test

Permutation tests were used to determine whether the number of overlapping genes is significantly higher than the expected number under the null model (i.e. by chance). The analysis was carried out using R. In brief, we first simulated the expected number of overlapping genes under the assumption that the induced responses and flower development are independent processes. To this end, we randomly selected a certain number of genes (same as the number of DEGs) from each of the background gene lists and calculated how many genes were found in both processes. We repeated this process 10,000 times (denoted as *N*). We then compared how many times (*n*) the simulated results were higher than the observed value and estimated the value of *p* with the following formula: *p*=(*n*+1) / (*N*+1). To determine the overlap between herbivory-induced genes and flowering-associated genes, we used all genes expressed in leaves and flowers of WT plants, respectively, as the background gene list. To determine the overlap between *LOXD*/*PS*-regulated genes in leaves and flowers, we used all genes expressed in the respective tissues of WT, *35S::PS* and *spr8* plants as the background dataset. The expression data and R-scripts are deposited on figshare.^4^

### Annotation of Putative Gene Functions

Functional annotation of tomato genes was performed by eggNOG-mapper v2^5^ (Huerta-Cepas et al., 2017, 2019) using the ITAG 3.2 protein sequences and predicted ORF protein sequences of the novel genes (resulting from transcript assembly) as input sequences, followed by the use of the R package AnnotationForge (v1.26.0; Carlson and Pagès, 2019). GO enrichment analysis was performed by R package clusterProfiler (v3.12.0; Yu et al., 2012) with all expressed genes as the background gene list. Enriched gene ontology (GO) terms with both value of *p* and value of *q*≤0.05 were selected. The top 10 enriched GO terms (at level four) were visualised as bar plots.

To identify genes involved in phytohormone biosynthesis and signalling, photosynthesis and secondary metabolism, we conducted a functional annotation with the reference proteins (ITAG 3.2) and predicted ORFs of the novel genes using Mercator4 v2.0^6^ (Schwacke et al., 2019). The genes involved in the different pathways were searched in MapMan (v3.6.0RC1; Thimm et al., 2004) based on the Mercator4 annotation and tomato metabolic pathway database.^7^

### Quantifying Phytohormones

Extraction was done similar as described by Bonaventure et al. (2011). In brief, approximately 10–15mg fine-ground freeze-dried plant material was extracted twice with ethyl acetate (Fisher Chemical). The supernatants were collected, combined and evaporated to dryness. After reconstitution in 70% methanol (Fisher Chemical) in water, the samples were analysed on an AB Sciex QTRAP 6500+ equipped with an Ion Drive^™^ Turbo V source with TurboIonSpray Probe, which was operated in negative ionisation mode multi-reaction-monitoring modus. Settings were as follows: curtain gas, 45; collision gas, high; IonSpray voltage, −4,500; temperature, 500°C; ion source gas 1, 50; ion source gas 2, 70; and entrance potential, −10. For quantification, we added 10ng D_6_-ABA (OlChemIm), 10ng D_5_-JA (Sigma-Aldrich) per sample as internal standard during the first extraction step. JA-Ile was quantified based on the D_5_-JA standard and an empirically determined conversion factor (m_JA-Ile_=0.274×m_D5-JA_).

### Quantifying Specialized Metabolites

Tissue was lyophilized for at least 24h and homogenised in GenoGrinder at 1,000 strokes per minute for 5min. Metabolites were extracted from approximately 2–20mg of lyophilized tissue in 1.5ml 80% methanol containing 27μg of umbelliferone (internal standard) for 2h and constant shaking. Extracts were centrifuged for 40min at 3,500rpm, and 100μl of supernatant was collected and analysed by LC-MS-QTOF. Extracts were analysed in AutoMSMS, negative mode using a Zorbax SB-C18 column (1.8μm, 2.1×100mm; Agilent). Quantification of α-tomatine was based on the mz1032.5385 (Rogachev and Aharoni, 2012) and the external standard.

### Gene Expression Validation

To validate the expression changes observed from RNA-seq, we selected 10 genes that are putatively related to the JA pathway or defences and measured their expression using qPCR. For each sample, ~1μg total RNA was used to synthesise first-strand cDNA using the GoScript^™^ Reverse Transcription Mix and Oligo(dT) kit (Promega, United States). The qPCRs reactions (in 10μl) were prepared according to the manufacturer’s instruction of UltraSYBR Mixture (High ROX; CWBIO, China). The qPCRs were performed on a StepOnePlus^™^ Real-Time PCR System (Applied Biosystems, United States) with following PCR parameters: predenaturation at 95°C for 10min, 40cycles of denaturation at 95°C for 15s and annealing/elongation at 60°C for 1min and melting curve was carried out in the end of each PCR with the default program to validate the specificity of primers. Primer sequences of the 10 selected genes are shown in Supplementary Table 1. Tomato *UBI3*, a common internal reference gene for analysing gene expression changes affected by the JA-signalling pathway or biotic stresses (Fowler et al., 2009), was used as a reference gene. The expression at stage 4 from WT was used for calibration. Relative expression values were calculated using the 2^−ΔΔCt^ method (Livak and Schmittgen, 2001). Three biological replicates (samples from three plants) and two technical replicates were used.

## Results

### Herbivory-Induced Transcriptomic Responses in Tomato Leaves

**Figure 1:**
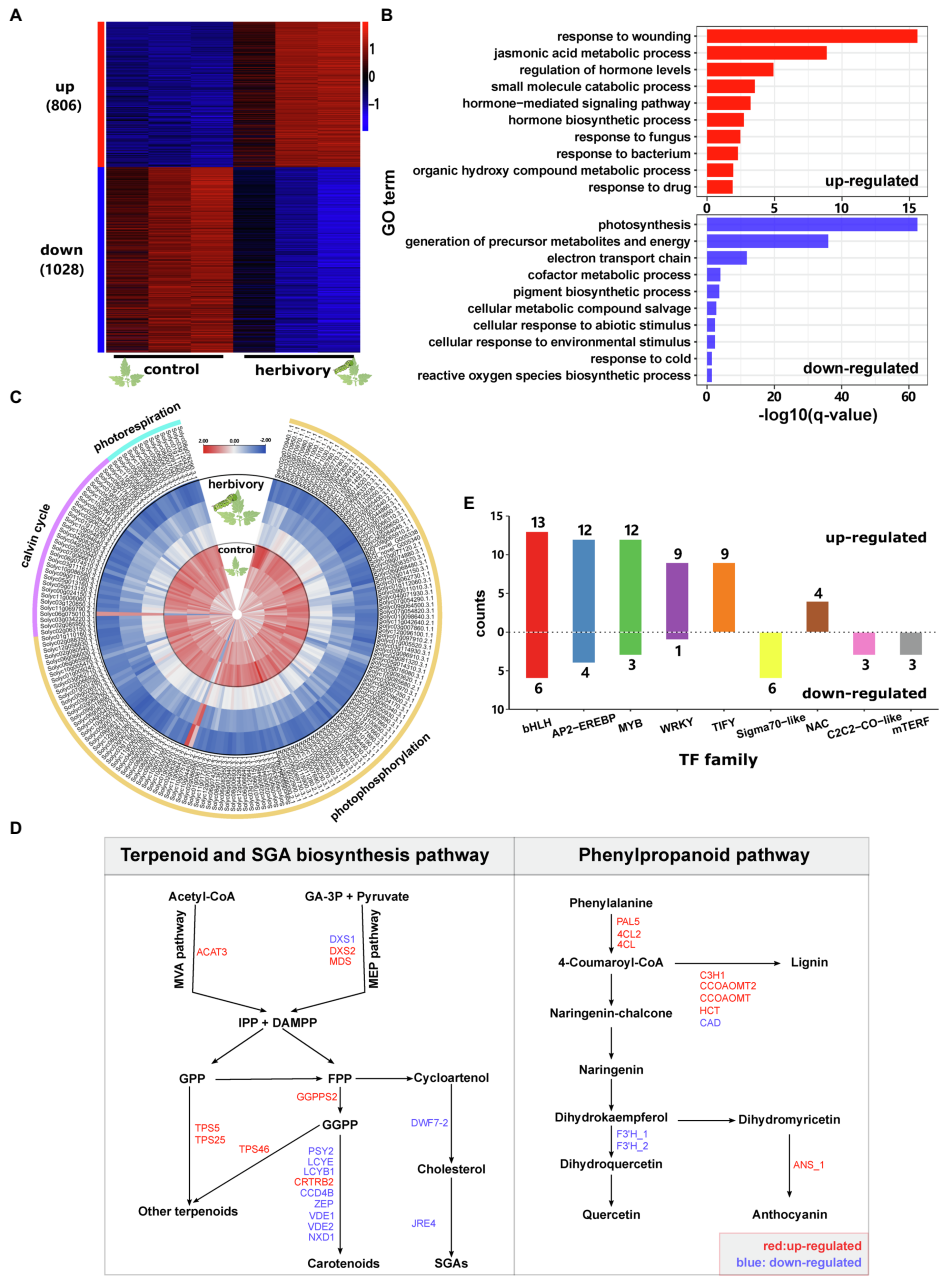
Herbivory-induced transcriptome changes in tomato leaves. **(A)** Heat map showing the expression patterns of herbivory-responsive genes in domesticated tomato. ‘up’ means upregulated by herbivory, and ‘down’ means downregulated by herbivory. The numbers in brackets are the corresponding gene numbers. ‘control’ and ‘herbivory’ represent control leaves and herbivore-infested leaves, respectively. Each treatment had three biological replicates. Gene expression abundance was log_10_-transformed and z-score normalised. **(B)** GO enrichment analysis for herbivory-induced (red column) and herbivory-repressed genes (blue column). Only the top 10 most enriched biological process terms are shown in the plots. **(C)** Heat map showing the expression patterns of genes involved in photosynthesis. The internal three circles represent the control leaves, while the external three circles represent herbivore-infested leaves. Each treatment had three replicates. Gene expression levels were log_10_-transformed and z-score normalised. **(D)** Herbivory-responsive genes involved in the terpenoid, SGA and phenylpropanoid pathways. Only genes showing significantly differential expression between the two treatments are shown. Gene names in blue represent genes downregulated by feeding, while gene names in red represent upregulated genes. IPP, isopentenyl diphosphate; DMAPP, dimethylallyl diphosphate; GA-3P, glyceraldehyde-3-phosphate; GPP, geranyl diphosphate; FPP, farnesyl diphosphate; and GGPP, geranylgeranyl diphosphate; for the full names of the indicated genes please refer to Supplementary Table 13. **(E)** Number of TFs which were up-or downregulated by herbivory sorted by TF family. Only TF families with at least three differentially expressed TFs were shown.

To investigate the herbivory-induced transcriptomic responses in tomato, we sequenced and compared transcriptomes of leaves from both control and herbivory-induced samples. In comparison with control samples, herbivory increased the transcript levels of 806 genes and decreased the levels of 1,028 genes (FDR≤0.05, fold change≥2; Figure 1A; Supplementary Table 2). Gene ontology (GO) enrichment analysis suggests that *M. sexta* feeding increased the expression of genes enriched in the functional terms: response to wounding, jasmonic acid metabolic process, regulation of hormone levels, small molecule catabolic process and hormone-mediated signalling pathway (Figure 1B). Genes that were suppressed by *M. sexta* feeding were enriched in the GO terms: photosynthesis, generation of precursor metabolites and energy, electron transport chain, cofactor metabolic process and pigment biosynthetic process (Figure 1B).

Annotating gene functions using MapMan revealed that *M. sexta* feeding increased the transcript levels of many phytohormone biosyntheses and signalling genes, in particular of the JA and ET pathway (Supplementary Table 3). In the JA biosynthesis pathway, four *LOXs*, three *AOSs*, *AOC*, *OPR3*, *OPCL1* and *ACX1A* were all significantly upregulated by herbivory (Supplementary Table 3). The same pattern was also found for several JA signalling genes: eight JASMONATE ZIM-domain (JAZ) genes, *MYC2*, and the targets of *MYC2*: *MTB1*, *MTB2* and *MTB3* (Supplementary Table 3). Consistently, herbivory also increased the levels of JA and JA-Ile in the leaves (Supplementary Figure 1) and upregulated JA-regulated defence genes, such as *CDI*, *TD2*, *LAP2*, *ARG2* and *PIs* (Supplementary Table 3). In the ethylene (ET) biosynthesis pathway, *M. sexta* feeding increased the transcript levels of *SAMS2*, *ACO1* and *ACO2* (Supplementary Table 3). A similar pattern was also found for the ET signalling genes, such as *ETR4*, *EIL1*, *EBF1/2* and several *ERFs* (Supplementary Table 3).

Among 181 photosynthesis-related genes that showed differential expression upon herbivory, 98.3% (178 out of 181) were downregulated by *M. sexta* feeding (Figure 1C). Consistently, many genes involved in carbohydrate metabolism were also downregulated (Supplementary Table 4).

We further performed permutation tests to identify metabolic pathways altered by herbivory. In addition to the changes in primary metabolism, in particular sugar, fatty acid and amino acid biosynthesis, several secondary metabolite pathways were also significantly altered (Table 1). In the terpenoid biosynthesis, *ACAT3*, *DXS2* and *MDS* were upregulated after herbivory (Figure 1D). These enzymes are involved in the mevalonate (MVA) and the methylerythritol phosphate (MEP) pathways, which are the key pathways to produce isopentenyl diphosphate (IPP) and dimethylallyl diphosphate (DMAPP) in plants (Lichtenthaler, 1999; Rohmer, 1999). In addition, a gene encoding geranylgeranyl pyrophosphate synthase (*GGPPS2*), as well as three terpene synthases (*TPS5*, *TPS25* and *TPS46*), also showed increased transcript levels in herbivory-induced leaves in comparison with control samples (Figure 1D). In the phenylpropanoid pathway, genes encoding phenylalanine ammonia lyase (*PAL5*), 4-coumarate-CoA ligase (*4CL2* and *4CL*), anthocyanidin synthase (*ANS_1*) and lignin biosynthesis genes, such as p-coumarate 3-hydroxylase (*C3H1*), caffeoyl-CoA 3-O-methyltransferase (*CCOAOMT* and *CCOAOMT2*) and hydroxycinnamoyl-CoA shikimate/quinate hydroxycinnamoyl transferase (*HCT*), were upregulated by insect feeding, while genes encoding for flavonoid-3′-hydroxylase (*F3′H_1* and *F3′H_2*) and cinnamyl-alcohol dehydrogenase (*CAD*) were downregulated after herbivory. Interestingly, *M. sexta* feeding decreased the expression of several genes involved in the biosynthesis of carotenoids and α-tomatine (Figure 1D).

**Table 1:** Permutation tests for the accumulation of herbivory-responsive DEGs in different metabolic pathways.

Pathway	Total number of genes per pathway	Number of observed DEGs	Number of DEGs expected by chance	*p* value
Aerobic respiration	192	1	10	1.000E+00
Benzoate biosynthesis	71	8	4	2.400E−02
CO_2_ fixation	68	23	3	9.999E−05
Fatty acid biosynthesis	171	19	9	8.999E−04
Fatty acid degradation	55	5	3	1.412E−01
Fatty acid derivative biosynthesis	88	18	4	9.999E−05
Glycan biosynthesis	180	11	9	2.990E−01
NAD metabolism	149	1	8	9.995E−01
Nitrogen-containing secondary compound biosynthesis	65	6	3	1.078E−01
Phenylpropanoid derivative biosynthesis	413	46	21	9.999E−05
Phospholipid biosynthesis	168	8	8	6.152E−01
Plant cell structures	302	26	15	6.099E−03
Plant hormone biosynthesis	354	34	18	2.000E−04
Polysaccharide biosynthesis	159	9	8	4.117E−01
Polysaccharide degradation	86	1	4	9.872E−01
Proteinogenic amino acid biosynthesis	231	27	12	9.999E−05
Proteinogenic amino acid degradation	134	17	7	4.000E−04
Purine nucleotide biosynthesis	105	5	5	6.142E−01
Pyrimidine nucleotide biosynthesis	75	5	4	3.194E−01
Reductant biosynthesis	59	2	3	8.023E−01
Sugar biosynthesis	219	35	11	9.999E−05
Sugar derivative biosynthesis	76	6	4	1.814E−01
Terpenoid biosynthesis	263	30	13	2.000E−04
Vitamin biosynthesis	112	4	6	8.215E−01

*Pathways with values of p less than 0.01 are highlighted in red*.

Transcription factors (TFs) are key elements for coordinated transcriptomic responses. In total, we found that 111 TFs from 29 families showed differential expression between control and *M. sexta*-attacked leaves (Figure 1E; Supplementary Table 5). Among them, bHLH, AP2-EREBP, MYB, WRKY and TIFY were the most frequent TF families. Among the differentially expressed bHLH TFs, 13 out of 19 were upregulated by herbivory, most of which are involved in regulating JA signalling. The TIFY family contains mostly JAZs, which are known targets but also suppressors of JA signalling. All DEGs from the TIFY family were upregulated by *M. sexta* feeding. The six DEGs from the Sigma70-like family were all suppressed by herbivory.

In summary, these results show that while feeding by *M. sexta* elicited JA and ET biosynthesis and signalling, as well as the biosynthesis of terpenoids and phenylpropanoids, it suppressed the photosynthesis and primary metabolisms in tomato leaves. The process is likely mediated by expression changes of a few key TF families.

### Transcriptomic Reprogramming During Flower Development in Tomato

**Figure 2:**
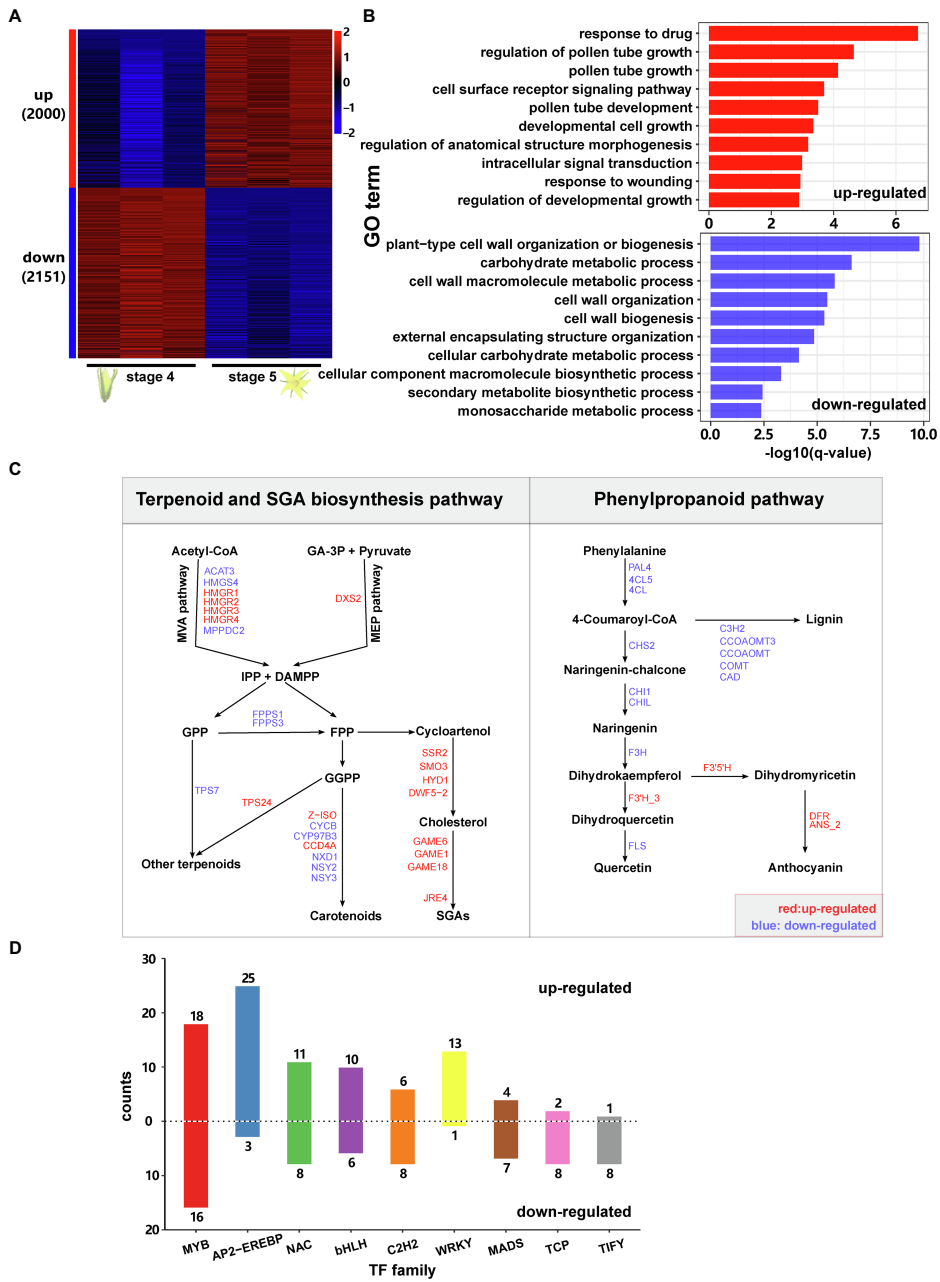
Transcriptomic reprogramming during flower development in tomato. **(A)** Heat map showing the expression patterns of flowering-associated genes in domesticated tomato. ‘up’ means upregulated during flower development, and ‘down’ means downregulated during flower development. The numbers in brackets are the corresponding gene numbers. For each stage, there were three replicates. Gene expression levels were log_10_-transformed and z-score normalised. **(B)** GO enrichment analysis for flowering-associated genes. Only the top ten most enriched GO terms are shown in the plots. **(C)** Expression patterns of flowering-associated genes involved in the terpenoid, SGA and phenylpropanoid pathways. Only genes showing significantly differential expression during flower development are shown in the figure. IPP, isopentenyl diphosphate; DMAPP, dimethylallyl diphosphate; GA-3P, glyceraldehyde-3-phosphate; GPP, geranyl diphosphate; FPP, farnesyl diphosphate; and GGPP, geranylgeranyl diphosphate; for the full names of the indicated genes please refer to Supplementary Table 13. **(D)** Number of TFs which were up-or downregulated during flower development sorted by TF family Only TF families with at least three differentially expressed TFs were shown.

To investigate transcriptomic changes during flower development, we collected and sequenced the transcriptomes of flower samples at two stages: stage 4 (flower bud shortly before opening) and stage 5 (fully opened flower). In total, 4,151 genes showed differential gene expression between the two stages, including 2,000 upregulated genes and 2,151 downregulated genes (Figure 2A; Supplementary Table 6). Among them, we found several previously reported genes involved in flower development and opening processes, such as the regulator of floral organ formation *SEPALLATA3* (*SEP3*), the gene involved in flower development *WUSCHEL* (*WUS*) and the stamen differentiation regulator *Tomato MADS-box gene 6* (*TM6*).

Gene ontology (GO) enrichment analysis showed that the upregulated genes are mainly enriched in the biological processes: regulation of pollen tube growth, cell surface receptor signalling pathway, pollen tube development, developmental cell growth and response to wounding (Figure 2B). The downregulated genes are most enriched in the GO terms: plant-type cell wall organisation or biogenesis, carbohydrate metabolic process, cell wall macromolecule metabolic process, cell wall organisation and secondary metabolite biosynthetic process (Figure 2B).

Functional annotation further revealed that during flower development, many DEGs are involved in phytohormone biosynthesis and signalling (Supplementary Table 7). For example, JA biosynthesis and signalling genes, as well as downstream defence genes, such as *LOXA*, *LOX11*, *AOS*, *AOC*, *OPCL1*, *JAZs*, *MTB2*, *CDI*, *ARG2*, *TD2*, *LAP2* and *PIs*, showed higher expression at stage 4 than at stage 5. In contrast, the important JA signalling regulator *MYC2* showed the opposite expression pattern. On the other hand, many ET biosynthesis and signalling genes, such as *ACSs*, *ACOs*, *ETRs*, *EIL2*, *EBFs* and some *ERFs*, were upregulated during flower development, showing much higher expression levels in open flowers than in flower buds (Supplementary Table 7).

We further performed permutation tests to identify the key metabolic pathways that were altered during flower development. The results show that not only the primary metabolism, in particular the biosynthesis of sugars, fatty acids and amino acids, but also several secondary metabolite pathways were significantly altered during flower development (Table 2). This includes the biosynthesis of phenylpropanoids, plant cell wall structures, polysaccharides and nitrogen-containing secondary compounds.

**Table 2:** Permutation tests for the accumulation of flowering-associated DEGs in different metabolic pathways.

Pathway	Total number of genes per pathway	Number of observed DEGs	Number of DEGs expected by chance	*p* value
Aerobic respiration	192	19	21	7.488E−01
Benzoate biosynthesis	71	12	8	9.369E−02
CO_2_ fixation	68	11	8	1.309E−01
Fatty acid biosynthesis	171	45	19	9.999E−05
Fatty acid degradation	55	6	6	5.928E−01
Fatty acid derivative biosynthesis	88	17	10	1.670E−02
Glycan biosynthesis	180	37	20	3.000E−04
NAD metabolism	149	11	17	9.516E−01
Nitrogen-containing secondary compound biosynthesis	65	14	7	1.200E−02
Phenylpropanoid derivative biosynthesis	413	58	46	3.990E−02
Phospholipid biosynthesis	168	41	19	9.999E−05
Plant cell structures	302	69	34	9.999E−05
Plant hormone biosynthesis	354	41	40	4.262E−01
Polysaccharide biosynthesis	159	34	18	5.000E−04
Polysaccharide degradation	86	22	10	2.000E−04
Proteinogenic amino acid biosynthesis	231	43	26	8.999E−04
Proteinogenic amino acid degradation	134	26	15	4.400E−03
Purine nucleotide biosynthesis	105	12	12	5.144E−01
Pyrimidine nucleotide biosynthesis	75	7	8	7.511E−01
Reductant biosynthesis	59	6	7	6.603E−01
Sugar biosynthesis	219	50	25	9.999E−05
Sugar derivative biosynthesis	76	22	9	9.999E−05
Terpenoid biosynthesis	263	37	29	8.439E−02
Vitamin biosynthesis	112	10	13	8.191E−01

*Pathways with values of p less than 0.01 are highlighted in red*.

Detailed analysis of these metabolic pathways revealed that genes involved in carbohydrate metabolism were downregulated during flower development, especially the genes for the biosynthesis of sucrose, starch and nucleotide sugar (Supplementary Table 4). Genes involved in steroidal glycoalkaloid (SGA) biosynthesis, including *SSR2*, *SMO3*, *HYD1*, *DWF5-2*, *GAMEs* and the key regulator *JRE4*, showed higher expression in open flowers than in flower buds (Figure 2C). In the phenylpropanoid biosynthesis, several genes involved in flavonoid biosynthesis, including *PAL4*, *4CL5*, *4CL*, *Chalcone synthase* (*CHS2*), *Chalcone isomerase* (*CHI1* and *CHIL*), *flavanone 3-hydroxylase* (*F3H*) and *flavonol synthase* (*FLS*), showed higher expression at stage 4 than at stage 5. The same expression pattern was observed for genes involved in lignin biosynthesis (Figure 2C). In contrast, *flavonoid-3′-hydroxylase* (*F3′H_3*), *flavonoid-3′5′-hydroxylase* (*F3′5′H*), *dihydroflavonol 4-reductase* (*DFR*) and *anthocyanidin synthase* (*ANS_2*) showed the opposite pattern (Figure 2C).

We further identified 259 TFs (from 43 families) that were differentially expressed between the two flowering stages (Figure 2D; Supplementary Table 8). Among them, more than 56% (146/259) were upregulated, showing higher expression levels at stage 5 (open flowers) than stage 4. The most frequent TF family was MYB, which contained 34 DEGs (18 upregulated and 16 downregulated genes). This included the previously characterised *MYB21*, which is required for flower opening and the development of reproductive organs (Niwa et al., 2018). Among other frequent TF families, AP2-EREBP, NAC, bHLH and WRKY contained more upregulated than downregulated genes, whereas C2H2, MADS, TCP and TIFY contained more downregulated genes. Interestingly, herbivory upregulated 9 TIFY genes, whereas 8 TIFY genes were downregulated during flower development.

Together, these results reveal that flower development is mostly associated with a downregulation of JA biosynthesis and upregulation of ET signalling, as well as a suppression of carbohydrate and phenylpropanoid metabolism and increasing SGA biosynthesis.

### The Shared Transcriptomic Responses in Flower Development and Leaf Herbivory

The similar metabolic responses found during flower development and leaf herbivory could be due to either a contribution of different genes from the same family or the same gene in these two seemingly distinct biological processes. Indeed, in the phenylpropanoid pathway, we observed different members of the same gene family that were involved in herbivory-induced responses and flower development. For example, in the anthocyanin biosynthesis *ANS_1* (Solyc10g076660) was upregulated by herbivory, while *ANS_2* (Solyc08g080040) was upregulated during flower development. Similar patterns were also found in the *PAL*, *4CL* and *C3H* families (Figures 1D, 2C). This suggests that some of the metabolic responses that are elicited both by herbivory and during flower development are achieved *via* independent regulation of different duplicated gene copies.

**Figure 3:**
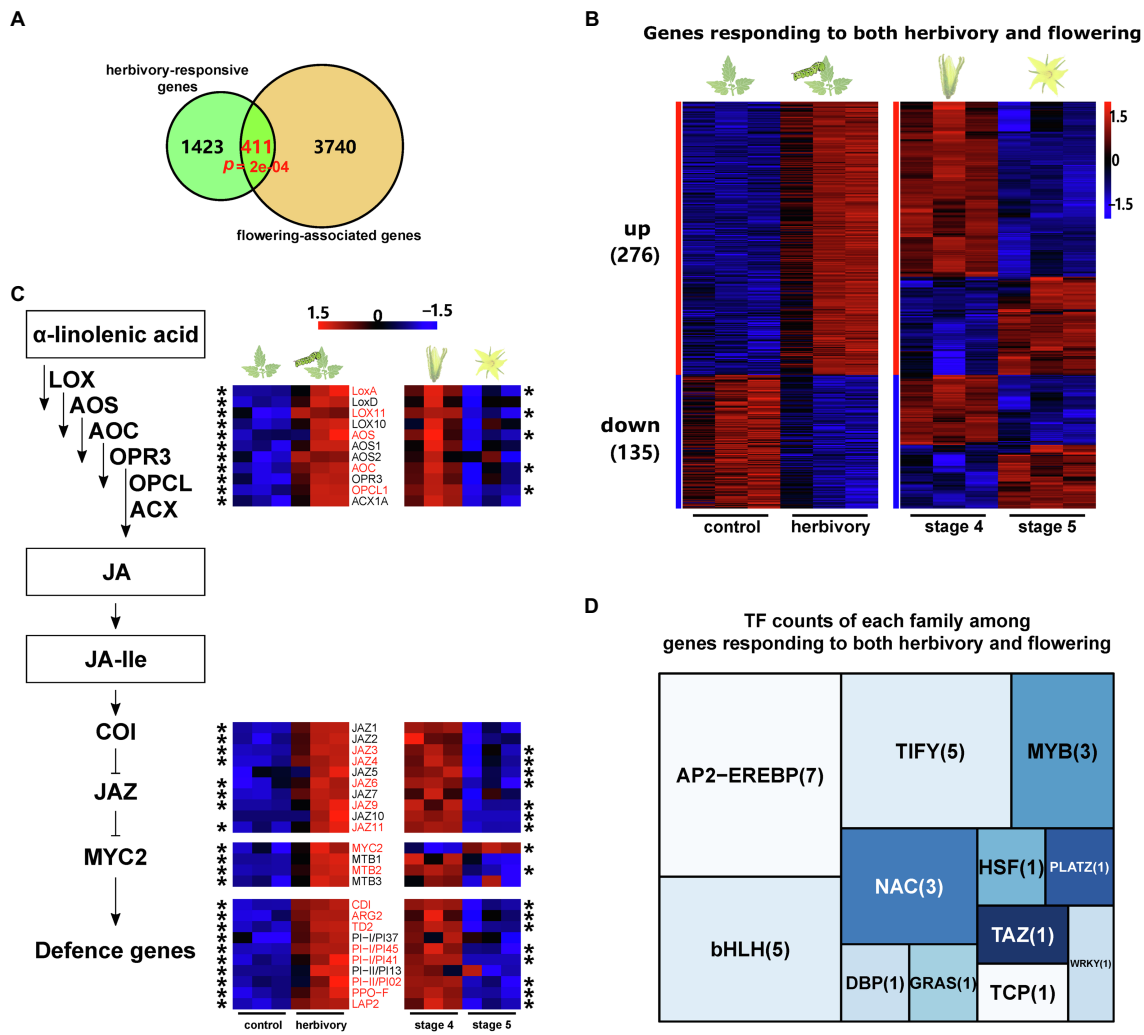
Shared transcriptomic responses during flower development and leaf herbivory. **(A)** Venn diagram showing the overlap between the flowering-associated genes (orange circle) and herbivory-responsive genes (green circle) in domesticated tomato. *p* denotes the probability to obtain the observed overlap by chance and was analysed by permutation test, *n*=10,000. **(B)** Heat maps showing the expression patterns of all shared DEGs in control and herbivore-infested leaves (left) and in flowers of stages 4 and 5 (right). ‘up’ means genes upregulated by herbivory, and ‘down’ means genes downregulated by herbivory. The numbers in brackets are the corresponding gene numbers. Gene expression levels were log_10_-transformed and z-score normalised. **(C)** Transcript response of genes related to JA biosynthesis, signalling and inducible plant defence after herbivory and during flower development. The heat maps show the expression patterns in control and herbivore-infested leaves (left), as well as in flowers of stages 4 and 5 (right). Only genes showing significant expression difference during herbivory or flower development are shown in the heat maps. Asterisks indicate that the expression of the respective gene was significantly different during the corresponding process. The genes highlighted in red significantly responded to both processes. Gene expression data were log_10_-transformed and z-score normalised. Each treatment had three replicates. **(D)** Tree map showing the number of TF genes responding to both herbivory and flower development, sorted by TF family.

A broader comparison revealed that among the 1,834 herbivory-induced genes, 411 were also found differentially expressed during flower development (Figure 3A; Supplementary Table 9). Permutation tests reveal that the observed overlap is significantly higher than by chance (*p*<0.001; 10,000 permutations). GO enrichment analysis showed that the overlapping DEGs are enriched in the GO terms: response to wounding and jasmonic acid metabolic process.

Among the 411 overlapping genes, 276 showed increased transcript levels after *M. sexta* feeding, of which around 2/3 were downregulated during flower development (Figure 3B). For example, genes in the JA biosynthesis (*LOXs*, *AOS*, *AOC* and *OPCL1*), JA signalling (*JAZ3*, *JAZ4*, *JAZ6*, *JAZ9*, *JAZ11* and *MTB2*) and JA-dependent defences (*ARG2*, *TD2*, *CDI*, *LAP2*, *PPO-F* and *PIs*) were upregulated by herbivory in leaves and downregulated during flower development (Figure 3C). The same pattern was also found for various genes of the MVA pathway (*ACAT3*), flavonoid biosynthesis (*4CL*) and lignin biosynthesis (*CCOAOMT*; Figures 1D, 2C). In contrast, several genes of the ET pathway (*ACO2*, EBF1/2, *ERF D2*, *ERF F4* and *ERF F5*) were upregulated both by *M. sexta* feeding and during flower development (Supplementary Tables 3 and 7).

The other 135 overlapping genes were suppressed by *M. sexta* feeding in leaves, and around half of them were also downregulated during flower development (Figure 3B). For example, several genes involved in carbohydrate metabolism and carotenoid biosynthesis were repressed by both herbivory and flower development (Figures 1D, 2C; Supplementary Table 4). Interestingly, *JAR4*, a key regulator of the SGA biosynthesis, was downregulated by herbivore feeding but upregulated during flower development.

More than 7% (30 out of 411) of the overlapping genes are transcription factors (Supplementary Table 10). Among them, the five most frequent families are: AP2-EREBP, bHLH, TIFY, MYB and NAC (Figure 3D). While most of the shared TFs (26 out of 30) were upregulated by *M. sexta* feeding in leaves, their expression patterns varied during flower development. More than half of the shared AP2-EREBP TFs (six out of seven) and bHLH TFs (three out of five) and all of the NAC TFs (three) were also upregulated during flower development. In contrast, all shared TIFY TFs (five) and MYB TFs (three) were suppressed in open flowers (stage 5).

### Signalling Networks Regulate Both Induced Defences and Flower Development

The correlated transcriptomic changes during flower development and herbivory-induced responses in leaves suggest that the same genes and signalling cascades regulate both processes. To test this hypothesis, we further compared the transcriptomes of WT plants and mutant plants in which herbivory-induced responses were genetically manipulated. We reasoned that if a signalling network is involved in regulating both processes, the genetic manipulation of its key elements will result in expression changes of similar genes in both processes. Here, we focus on the comparisons between WT and two mutant/transgenic plants: *spr8* and *35S::PS*. In *spr8* plants, the expression of *LOXD* (a JA biosynthesis gene) is altered due to a mutation, which results in significantly lower levels of herbivory-induced JA and JA-Ile than in WT plants (Yan et al., 2013; Supplementary Figure 2). In *35S::PS* plants, *PS* is overexpressed by a 35S promoter from the Cauliflower mosaic virus, resulting in high levels of endogenous JA and JA-regulated defence responses, even without herbivore attacks (Chen et al., 2006).

**Figure 4:**
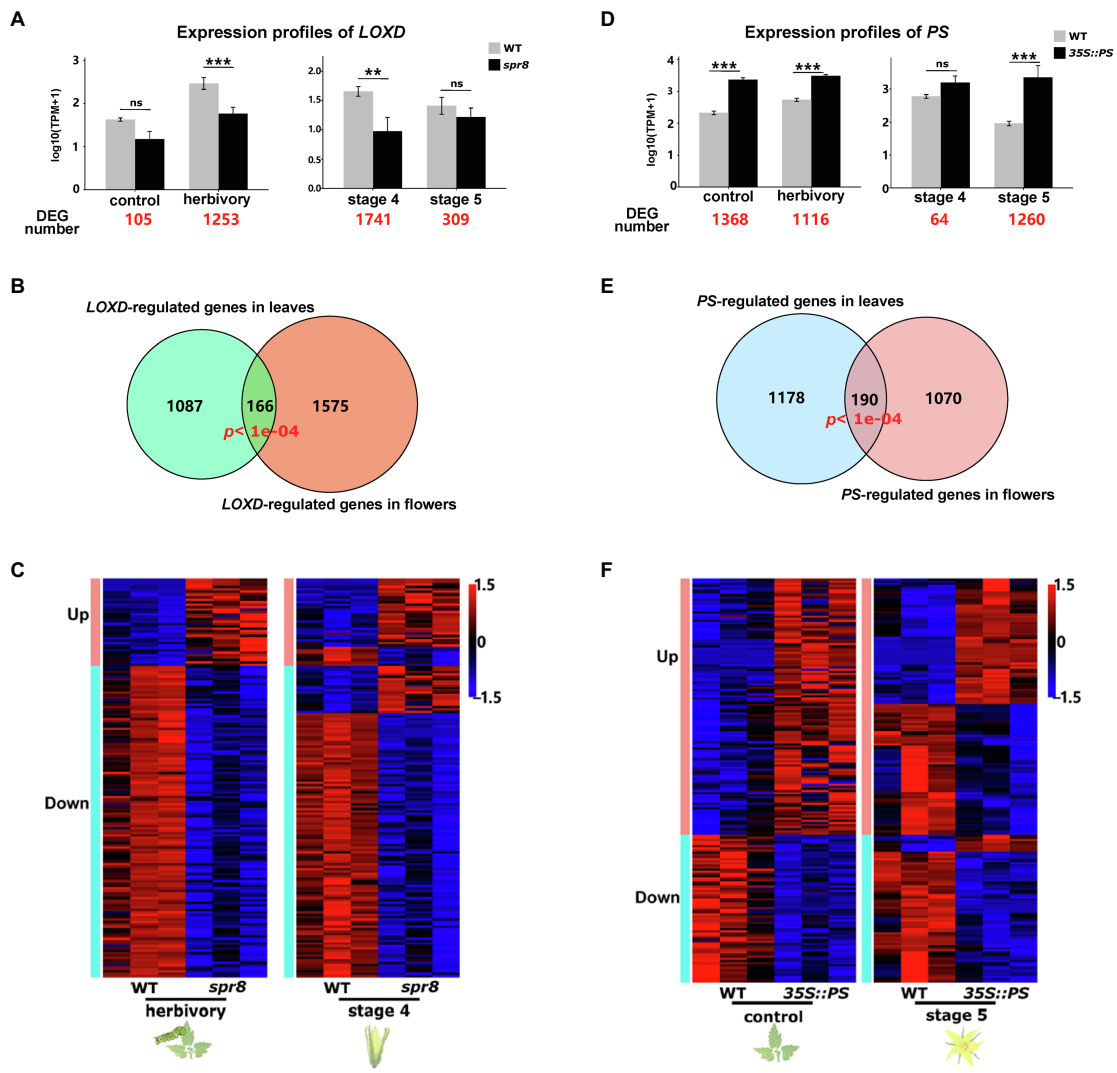
The signalling machinery required for herbivory-induced responses is partially also involved in flower development. **(A)** Expression profile of *LOXD* in herbivore-infested leaves and untreated controls, as well as during flower development (stages 4 and 5) of WT (grey bars) and *spr8* plants (black bars). The numbers highlighted in red represent the number of DEGs between WT and *spr8* plants within each treatment or stage. **(B)** Venn diagram showing the overlap between *LOXD*-regulated DEGs observed in herbivory-infested leaves and during flower development at stage 4. *p* denotes the probability to obtain the observed overlap by chance and was analysed by permutation test, *n*=10,000. **(C)** Heat maps showing the expression patterns of *LOXD*-regulated genes in both herbivory-infested leaves and during flower development (stage 4) in WT and *spr8* plants. **(D)** Expression profile of *PS* in herbivore-infested leaves and untreated controls, as well as during flower development (stages 4 and 5) of WT (grey bars) and *35S::PS* plants (black bars). The numbers highlighted in red represent the number of DEGs between WT and *35S::PS* plants within each treatment or stage. **(E)** Venn diagram showing the overlap between *PS*-regulated DEGs observed in control leaves and during flower development at stage 5. *p* denotes the probability to obtain the observed overlap by chance and was analysed by permutation test, *n*=10,000. **(F)** Heat maps showing the expression patterns of *PS*-regulated genes in both control leaves and during flower development (stage 5). Asterisks indicate differential expression of a gene between different genotypes in the same treatment or stage in **(A)** and **(D)**. Based on the criterion of DEGs (see Section ‘Materials and Methods’), ^**^ denotes *p*≤0.01 and ^***^ denotes *p*≤0.001; ns, not significant. Error bars indicate standard errors.

First, we compared both the leaf and flower transcriptomes of WT plants and *spr8* mutants. Between leaves from WT and *spr8* plants, only 105 genes were differentially expressed in control samples (without herbivory), while 1,253 genes were differentially expressed in herbivory-induced samples (Figure 4A), which included most of the well-studied herbivory-induced defence responses, such as *TD2*, *ARG2* and *PIs*. This is consistent with a previous study showing that the herbivory-induced *LOXD* expression and JA signalling are impaired in the *spr8* mutant (Yan et al., 2013). Functional enrichment analysis indicated that these 1,253 genes are enriched in the GO processes of: photosynthesis, generation of precursor metabolites and energy and response to wounding (Supplementary Figure 3A). Between flowers of WT and *spr8* plants, the transcriptomes differed most at stage 4, where transcript levels of *LOXD* were significantly different (Figure 4A). In total, we found 1,741 DEGs at this stage that are involved in pollen tube growth, response to wounding and pollination (Figure 4A, Supplementary Figure 3B). Among them, 166 were also found differentially expressed in herbivory-induced leaves (Figure 4B; Supplementary Table 11). Permutation tests showed that the observed number of genes (166) found to be differentially expressed between WT and *spr8* plants both in leaves and flowers is significantly higher than expected by chance (*p*<0.001; 10,000 permutations). These 166 genes are primarily enriched in the biological process response to wounding and contain mostly well-characterised herbivory-induced JA-signalling and anti-herbivore defence genes, such as *LOXs*, *AOS*, *JAZs*, *TD2*, *ARG2* and *LAP2*. In addition, the gene expression changes between WT and *spr8* plants in leaves and flowers were highly similar. For example, most genes downregulated in herbivory-induced leaves of *spr8* plants were also downregulated in the flowers of *spr8* plants (Figure 4C).

Second, we compared the leaf and flower transcriptomes of WT and *35S::PS* plants. Comparing WT and *35S::PS* plants revealed 1,368 and 1,116 DEGs in control and herbivory-induced leaves, respectively (Figure 4D). This is consistent with the fact that the differences in transcript levels of *PS* between WT and *35S::PS* plants were larger under control conditions than under herbivory (Figure 4D). GO analysis showed that the 1,368 genes are highly related to photosynthesis, generation of precursor metabolites and energy and pigment biosynthetic process (Supplementary Figure 3C). In flowers, the largest transcriptomic difference between WT and *35S::PS* plants was found at developmental stage 5 (open flowers), where *PS* showed significantly different transcript levels (Figure 4D). In total, 1,260 genes were differentially expressed, which are enriched in GO terms of: secondary metabolite biosynthetic process, regulation of hormone levels and response to nitrogen compound (Supplementary Figure 3D). Among them, 190 were differentially expressed in leaves (under control conditions; Figure 4E; Supplementary Table 12). Permutation tests showed that the observed number of genes (190) that were found both differentially expressed in leaves and flowers of WT and *35S::PS* plants is significantly higher than expected by chance (*p*<0.001; 10,000 permutations). These 190 genes are mostly involved in photosynthesis and herbivory-induced defence responses, such as *PIs* and *LAP*. While half of the genes activated by the over-expression of *PS* in leaves also showed increased expression in flowers, most genes that were suppressed by over-expression of *PS* in leaves also showed decreased transcript levels in flowers (Figure 4F).

To validate the results from RNA-seq, we further measured the expression of 10 selected genes using qPCR, in both WT and s*pr8* flowers in the stage 4 and 5. The expression patterns of the selected genes observed from RNA-seq are overall highly consistent with qPCR (Supplementary Figure 4).

Together, these results reveal that herbivory-induced responses in leaves and flower development are partially regulated by the same signalling cascades.

### Diffuse Coevolution of Defensive Metabolites in Tomato Leaves and Flowers

**Figure 5:**
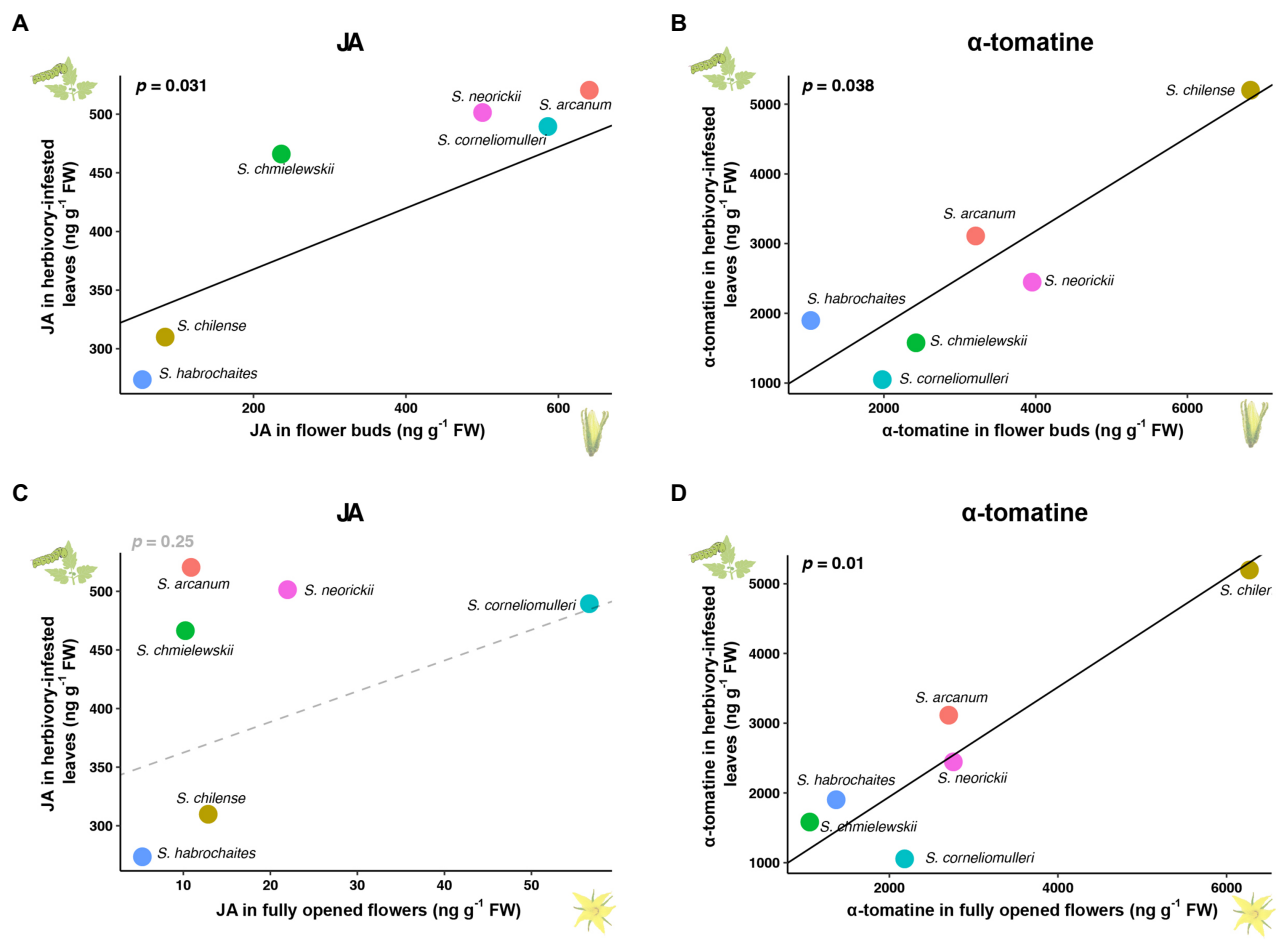
Diffuse coevolution of JA and α-tomatine in tomato leaves and flowers. **(A,C)** Correlated changes of JA between herbivory-infested leaves and flower buds **(A)** or fully opened flowers **(C)** among six wild tomato species. **(B,D)** Correlated changes of α-tomatine between herbivory-infested leaves and flower buds **(B)** or fully opened flowers **(D)** among six wild tomato species. Considering the phylogenetic relationship, correlated variations were analysed using a generalised least squares method with a phylogenetic tree based on the phylogeny of *Solanum* sect. *Lycopersicon* from Pease et al. (2016).

The shared signalling network predicts that defensive metabolites can show diffuse coevolution in flower development and herbivory-induced responses. To test this prediction, we analysed the concentration of several specialised metabolites and phytohormones (α-tomatine, kaempferol, rutin, JA, JA-Ile and abscisic acid) in six closely related wild tomato species. Among these wild tomatoes, only JA and α-tomatine, two key defence-related metabolites in tomato, showed correlated variations between flowers and herbivory-induced leaves (*p*<0.05; after correction for phylogeny; Figure 5; Supplementary Figure 5). While α-tomatine showed a significant correlation between the induced leaves and flowers in both developmental stages, JA only co-varied between the induced leaves and flower buds. Interestingly, while previous studies have shown that α-tomatine is regulated by JA (Cárdenas et al., 2016; Abdelkareem et al., 2017; Montero-Vargas et al., 2018; Nakayasu et al., 2018), no correlation was found between the levels of herbivory-induced JA and α-tomatine (*p*>0.1), suggesting additional genetic mechanisms are also involved in herbivory-induced α-tomatine changes.

## Discussion

Both herbivory-induced responses in leaves and flower development are highly relevant processes for plant reproduction and fitness. However, they were often studied in isolation. Using a comparative transcriptomics approach, we found that the two seemly distinct biological processes require a similar transcriptional regulation: modulation of JA signalling, suppression of primary metabolisms and reprogramming of secondary metabolisms. While in some pathways different duplicated gene copies specifically responded to one or the other process, further genetic manipulations revealed that herbivory-induced responses in leaves and flower development are partially regulated by shared signalling networks. Together with correlated changes in key defence metabolites among six closely related tomato species, these results suggest that floral traits and herbivory-induced defences likely have evolved under diffuse selection due to pleiotropy.

In tomato leaves, *M. sexta* herbivory increased the transcript levels of genes involved in the JA and ET pathways (Supplementary Table 3), as well as the secondary metabolism, but decreased the transcript levels of genes involved in primary metabolism. This pattern is consistent with herbivory-induced responses in wild tobacco (*Nicotiana attenuata*; Diezel et al., 2009), *Arabidopsis thaliana* (De Vos et al., 2005; Ehlting et al., 2008), cotton (*Gossypium hirsutum*; Huang et al., 2015), maize and rice (Qi et al., 2018).

The elicitation of JA and ET biosynthesis and signalling can lead to reprogramming of the specialised metabolism, which is a widespread phenomenon in plants (Bennett and Wallsgrove, 1994; War et al., 2012; Fürstenberg-Hägg et al., 2013; Aljbory and Chen, 2018). Some of these induced responses result in increased plant defences. For example, in tomato, the increased expression of the anti-digestive proteins (such as PIs and TDs) can reduce the digestive capacity of herbivores (Chung and Felton, 2011), and the increased expression of terpene synthase genes (*TPS5*, *TPS25* and *TPS46*) is involved in the release of volatiles that can attract their natural enemies (Figure 1D; Paudel et al., 2019). Interestingly, although SGAs are known as effective defence compounds against biotic attackers because of their cytotoxic and anti-nutritional properties (Roddick, 1996; Milner et al., 2011), most of the SGA biosynthesis genes were not altered by herbivory in our experiments (except *DWF7-2* and *JRE4*, which were downregulated by feeding; Figure 1D). In the tomato *jre4* mutant, SGA accumulation is decreased and susceptibility to *Spodoptera littoralis* larvae is increased (Nakayasu et al., 2018). Because *JRE4* is JA-inducible, it is reasonable to assume that *JRE4* is part of the herbivory-induced plant defence responses (Nakayasu et al., 2018). However, our data show that the expression of *JRE4* was suppressed by *M. sexta* feeding, which could be due to the induction of the ET pathway that can suppress the JA-induced *JRE4* expression in tomato leaves (Nakayasu et al., 2018).

In addition to chemical defences, herbivore attack can also alter the cell wall metabolism. For example, in maize, the attack by *Sesamia nonagrioides* upregulates cell wall reorganisation and biogenesis, which is accompanied by increased lignin content (Rodríguez et al., 2012). Consistently, *M. sexta*-infested tomato leaves also show increased expression levels of genes involved in the phenylpropanoid pathway and lignin biosynthesis (*PAL5*, *4CL*, *4CL2*, *C3H1*, *CCOAOMT*, *CCOAOMT2* and *HCT*; Figure 1D).

We found a majority of genes involved in carbohydrate metabolism and photosynthesis that showed a significantly decreased expression in herbivore-attacked leaves (Figure 1C; Supplementary Table 4). Herbivory-induced suppression of photosynthesis and carbohydrate metabolism is common among different plant taxa after attack by herbivores from different feeding guilds (Halitschke et al., 2003; Voelckel et al., 2004; Voelckel and Baldwin, 2004; Zhu-salzman et al., 2004; Ralph et al., 2006; Bilgin et al., 2010), with the exception of mirid bugs feeding on wild tobacco (Halitschke et al., 2011). Similarly, herbivory and artificially increased endogenous jasmonate levels can lead to decreased carbohydrate concentrations (Babst et al., 2005; Machado et al., 2013, 2015, 2017; Ferrieri et al., 2015). Therefore, our results fall in line with previous observations regarding changes in carbohydrate metabolism following herbivory.

During flower development, we also observed transcriptional changes of genes involved in the JA and ET pathways, the primary metabolism and the secondary metabolism. The observed changes in JA signalling during flower development are consistent with previous studies that showed the importance of JA signalling for flower development (Yuan and Zhang, 2015; Huang et al., 2017). Previous studies additionally showed that JA and JA-Ile levels peak at the bud stage and gradually decline to basal levels in fully opened flowers (Dobritzsch et al., 2015; Niwa et al., 2018). Consistently, we observed a higher expression of JA biosynthesis genes at stage 4 than at stage 5 (Figure 3C; Supplementary Table 7). In addition to JA, ET also plays a fundamental role in flower development in *Arabidopsis*, rice, China rose (*Hibiscus rosa sinensis*) and tomato (Iqbal et al., 2017). We observed that the transcript levels of ET biosynthesis and signalling genes were higher in fully opened flowers than in flower buds (Supplementary Table 7). This is consistent with the fact that ET regulates flower senescence and fruit ripening (Lanahan et al., 1994; Tieman et al., 2000; Alexander and Grierson, 2002; Shinozaki et al., 2015).

Flower development involves several metabolic shifts. First, flower development requires secondary cell wall biogenesis (Shalit-Kaneh et al., 2019). Our analysis showed that the expression of genes involved in lignin biosynthesis, including *PAL4*, *4CL*, *4CL5*, *C3H2*, *CCOAOMT*, *CCOAOMT3*, *COMT* and *CAD*, was suppressed in fully opened flowers in comparison with flower buds (Figure 2C). Similarly, in snapdragon flowers, downregulated genes during petal development were notably enriched for enzymes of secondary cell wall biogenesis (Muhlemann et al., 2012). Second, flower development involves petal expansion, which involves hydrolysis of stored carbohydrates (Bieleski, 1993; Bieleski et al., 2000; Vergauwen et al., 2000; Yap et al., 2008). This might explain the observed higher expression of genes involved in starch and sucrose degradation in fully opened flowers (stage 5) compared to flower buds (stage 4; Supplementary Table 4). Third, flower development also involves the increased biosynthesis of pigments and volatiles that are critical for pollinator attraction. We found that the expression of genes involved in late steps of carotenoids biosynthesis (e.g. *CYCB*, *CYP97B3*, *NXD1*, *NSY2* and *NSY3*) was mostly suppressed in fully opened flowers (Figure 2C), while the expression of genes involved in anthocyanin biosynthesis, including *F3′5′H*, *DFR* and *ANS_2*, was increased. In addition, a key gene in flavonol biosynthesis, *FLS*, was suppressed in fully opened flowers (Figure 2C). This suggests that the metabolism of flavonoids in tomato flowers shifts towards the production of anthocyanins during the opening process, which is similar to the pattern observed in *Eustoma grandifloru* (Kawabata et al., 2009). Interestingly, the expression of *TPS7*, a monoterpene synthase that mainly catalyses the formation of β-myrcene and limonene in tomato (Falara et al., 2011; Zhou and Pichersky, 2020), was reduced in fully opened flowers in comparison with flower buds. Surprisingly, most of the SGAs biosynthesis genes were upregulated during flower development, which might explain why the concentration of SGAs is higher in flowers than in other tissues (Friedman and Levin, 1995). The higher abundance of SGAs in flowers might also be related to the phytotoxicity of the intermediate metabolites in SGA biosynthesis (Itkin et al., 2011).

Although herbivory-induced responses and flower development are two distinct biological processes, they partially share the same regulatory mechanisms. We identified 411 genes that were affected by both herbivory and flower development (Figure 3A). Among them, many genes are associated with phytohormone biosynthesis and signalling (e.g. JA and ET), carbohydrate metabolism, the MVA and MEP pathways, as well as the phenylpropanoid pathway. Further, the manipulation of key regulators (*LOXD* and *PS*) of the herbivory-induced response confirmed that the same signalling machinery is involved in both processes. The shared signalling machinery of both the herbivory-induced responses and flower development suggests diffuse coevolution of plant defence and floral signals (Jacobsen and Raguso, 2018; Rusman et al., 2019; Ramos and Schiestl, 2020). Interestingly, in the phenylpropanoid pathway, we observed that different duplicated gene copies specifically responded to one or the other process (Figures 1D, 2C), suggesting that plants can also escape from pleiotropic effects *via* gene duplications. However, a significantly higher number of genes than expected by chance is shared by both induced defence responses and flower development, indicating that some of the pleiotropic effects are likely adaptive. However, it is worth noticing that the observed pleiotropy is likely an underestimate. Due to the use of complete flower buds and flowers, we likely have missed the gene expression changes that are specific in different floral organs, which were marked due to pooling effects. Future studies that analyse the transcriptome of each floral organ will provide further insights into shared pleiotropy between induced responses and flower development.

Consistent with the transcriptomic data, we found the content of JA and α-tomatine was correlated between flower buds and herbivory-induced leaves among closely related wild tomato species (Figures 5A,B), suggesting that herbivory-induced defences and floral traits coevolved due to the same molecular regulatory networks. However, the sample size in this study is relatively small, which might limit the power to detect other co-evolving traits. Future studies that include large sample size and systematically compare metabolomes and transcriptomes between flowers and induced leaves will provide a better understanding on the extent to which diffuse coevolution shapes the evolution of defences and floral signals in nature.

## Data Availability Statement

The datasets presented in this study can be found in online repositories. The names of the repository/repositories and accession number(s) can be found at: https://www.ncbi.nlm.nih.gov/, PRJNA600385.

## Author Contributions

SX conceived the study. SX, RZ and YS planned the research. LK, YW, MS, JF, TS and HP performed the experiment and analysed the data. SX, RZ, CD and YS contributed the resources. SX and LK wrote the manuscript. All authors contributed to the article and approved the submitted version.

## Funding

This research was supported by the National Natural Science Foundation of China (31770474 and 31870361), the Natural Science Foundation of Fujian Province (2020J02030), International cooperation and exchange program at the Fujian Agriculture and Forestry University (KXGH17018) the Center for Adaptation to a Changing Environment (ACE) at ETH Zürich and the University of Münster.

## Conflict of Interest

The authors declare that the research was conducted in the absence of any commercial or financial relationships that could be construed as a potential conflict of interest.

## Publisher’s Note

All claims expressed in this article are solely those of the authors and do not necessarily represent those of their affiliated organizations, or those of the publisher, the editors and the reviewers. Any product that may be evaluated in this article, or claim that may be made by its manufacturer, is not guaranteed or endorsed by the publisher.

## Footnotes

^1^ https://www.ricoter.ch

^2^ https://www.hauert.com

^3^ https://submit.ncbi.nlm.nih.gov/subs/sra/

^4^ https://figshare.com/s/e4e9a2b64be4a7d91282

^5^ http://eggnog-mapper.embl.de/

^6^ https://www.plabipd.de/portal/web/guest/mercator4

^7^ https://pmn.plantcyc.org/TOMATO/class-tree?object=Pathways

## References

[ref1] Abdelkareem A. Thagun C. Nakayasu M. Mizutani M. Hashimoto T. Shoji T. (2017). Jasmonate-induced biosynthesis of steroidal glycoalkaloids depends on COI1 proteins in tomato. Biochem. Biophys. Res. Commun. 489, 206–210. doi: 10.1016/j.bbrc.2017.05.132, PMID: 28554842

[ref2] Agrawal A. F. Stinchcombe J. R. (2009). How much do genetic covariances alter the rate of adaptation? Proc. R. Soc. B Biol. Sci. 276, 1183–1191. doi: 10.1098/rspb.2008.1671, PMID: 19129097 PMC2679087

[ref3] Alexander L. Grierson D. (2002). Ethylene biosynthesis and action in tomato: a model for climacteric fruit ripening. J. Exp. Bot. 53, 2039–2055. doi: 10.1093/jxb/erf072, PMID: 12324528

[ref4] Aljbory Z. Chen M. S. (2018). Indirect plant defense against insect herbivores: a review. Insect Sci. 25, 2–23. doi: 10.1111/1744-7917.12436, PMID: 28035791

[ref5] Al-Zahrani W. Bafeel S. O. El-Zohri M. (2020). Jasmonates mediate plant defense responses to *Spodoptera exigua* herbivory in tomato and maize foliage. Plant Signal. Behav. 15:e1746898. doi: 10.1080/15592324.2020.1746898, PMID: 32290765 PMC7238883

[ref6] Babst B. A. Ferrieri R. A. Gray D. W. Lerdau M. Schlyer D. J. Schueller M.. (2005). Jasmonic acid induces rapid changes in carbon transport and partitioning in *Populus*. New Phytol. 167, 63–72. doi: 10.1111/j.1469-8137.2005.01388.x, PMID: 15948830

[ref7] Bennett R. N. Wallsgrove R. M. (1994). Secondary metabolites in plant defence mechanisms. New Phytol. 127, 617–633. doi: 10.1111/j.1469-8137.1994.tb02968.x, PMID: 33874382

[ref8] Bieleski R. L. (1993). Fructan hydrolysis drives petal expansion in the ephemeral daylily flower. Plant Physiol. 103, 213–219. doi: 10.1104/pp.103.1.213, PMID: 12231928 PMC158965

[ref9] Bieleski R. Elgar J. Heyes J. (2000). Mechanical aspects of rapid flower opening in Asiatic lily. Ann. Bot. 86, 1175–1183. doi: 10.1006/anbo.2000.1291

[ref10] Bilgin D. D. Zavala J. A. Zhu J. Clough S. J. Ort D. R. Delucia E. H. (2010). Biotic stress globally downregulates photosynthesis genes. Plant Cell Environ. 33, 1597–1613. doi: 10.1111/j.1365-3040.2010.02167.x, PMID: 20444224

[ref11] Bonaventure G. Schuck S. Baldwin I. T. (2011). Revealing complexity and specificity in the activation of lipase-mediated oxylipin biosynthesis: a specific role of the *Nicotiana attenuata* GLA1 lipase in the activation of jasmonic acid biosynthesis in leaves and roots. Plant Cell Environ. 34, 1507–1520. doi: 10.1111/j.1365-3040.2011.02348.x, PMID: 21554327

[ref12] Bosch M. Wright L. P. Gershenzon J. Wasternack C. Hause B. Schaller A.. (2014). Jasmonic acid and its precursor 12-oxophytodienoic acid control different aspects of constitutive and induced herbivore defenses in tomato. Plant Physiol. 166, 396–410. doi: 10.1104/pp.114.237388, PMID: 25073705 PMC4149723

[ref13] Cárdenas P. D. Sonawane P. D. Pollier J. Vanden Bossche R. Dewangan V. Weithorn E.. (2016). GAME9 regulates the biosynthesis of steroidal alkaloids and upstream isoprenoids in the plant mevalonate pathway. Nat. Commun. 7:10654. doi: 10.1038/ncomms10654, PMID: 26876023 PMC4756317

[ref14] Carlson M. Pagès H. (2019). AnnotationForge: tools for building SQLite-based annotation data packages. R package version 1.26.0.

[ref15] Chen H. (2018). VennDiagram: generate high-resolution venn and euler plots. R package version 1.6.20.

[ref16] Chen C. Chen H. Zhang Y. Thomas H. R. Frank M. H. He Y.. (2020). TBtools: an integrative toolkit developed for interactive analyses of big biological data. Mol. Plant 13, 1194–1202. doi: 10.1016/j.molp.2020.06.009, PMID: 32585190

[ref17] Chen H. Jones A. D. Howe G. A. (2006). Constitutive activation of the jasmonate signaling pathway enhances the production of secondary metabolites in tomato. FEBS Lett. 580, 2540–2546. doi: 10.1016/j.febslet.2006.03.070, PMID: 16647069

[ref18] Chen H. Wilkerson C. G. Kuchar J. A. Phinney B. S. Howe G. A. (2005). Jasmonate-inducible plant enzymes degrade essential amino acids in the herbivore midgut. Proc. Natl. Acad. Sci. U. S. A. 102, 19237–19242. doi: 10.1073/pnas.0509026102, PMID: 16357201 PMC1323180

[ref19] Chung S. H. Felton G. W. (2011). Specificity of induced resistance in tomato against specialist lepidopteran and coleopteran species. J. Chem. Ecol. 37, 378–386. doi: 10.1007/s10886-011-9937-0, PMID: 21455676

[ref20] Chung H. S. Koo A. J. K. Gao X. Jayanty S. Thines B. Jones A. D.. (2008). Regulation and function of Arabidopsis *JASMONATE ZIM*-domain genes in response to wounding and herbivory. Plant Physiol. 146, 952–964. doi: 10.1104/pp.107.115691, PMID: 18223147 PMC2259048

[ref21] De Geyter N. Gholami A. Goormachtig S. Goossens A. (2012). Transcriptional machineries in jasmonate-elicited plant secondary metabolism. Trends Plant Sci. 17, 349–359. doi: 10.1016/j.tplants.2012.03.001, PMID: 22459758

[ref22] De Moraes C. M. Mescher M. C. Tumlinson J. H. (2001). Caterpillar-induced nocturnal plant volatiles repel conspecific females. Nature 410, 577–580. doi: 10.1038/35069058, PMID: 11279494

[ref23] De Vos M. Van Oosten V. R. Van Poecke R. M. P. Van Pelt J. A. Pozo M. J. Mueller M. J.. (2005). Signal signature and transcriptome changes of *Arabidopsis* during pathogen and insect attack. Mol. Plant-Microbe Interact. 18, 923–937. doi: 10.1094/MPMI-18-0923, PMID: 16167763

[ref24] Diezel C. von Dahl C. C. Gaquerel E. Baldwin I. T. (2009). Different lepidopteran elicitors account for cross-talk in herbivory-induced phytohormone signaling. Plant Physiol. 150, 1576–1586. doi: 10.1104/pp.109.139550, PMID: 19458114 PMC2705021

[ref25] Dobritzsch S. Weyhe M. Schubert R. Dindas J. Hause G. Kopka J.. (2015). Dissection of jasmonate functions in tomato stamen development by transcriptome and metabolome analyses. BMC Biol. 13:28. doi: 10.1186/s12915-015-0135-3, PMID: 25895675 PMC4443647

[ref26] Dudareva N. Klempien A. Muhlemann J. K. Kaplan I. (2013). Biosynthesis, function and metabolic engineering of plant volatile organic compounds. New Phytol. 198, 16–32. doi: 10.1111/nph.12145, PMID: 23383981

[ref27] Durrant M. Boyer J. Zhou W. Baldwin I. T. Xu S. (2017). Evidence of an evolutionary hourglass pattern in herbivory-induced transcriptomic responses. New Phytol. 215, 1264–1273. doi: 10.1111/nph.14644, PMID: 28618009

[ref28] Ehlting J. Chowrira S. G. Mattheus N. Aeschliman D. S. Arimura G. I. Bohlmann J. (2008). Comparative transcriptome analysis of *Arabidopsis thaliana* infested by diamond back moth (*Plutella xylostella*) larvae reveals signatures of stress response, secondary metabolism, and signalling. BMC Genomics 9:154. doi: 10.1186/1471-2164-9-154, PMID: 18400103 PMC2375910

[ref29] Erb M. Reymond P. (2019). Molecular interactions between plants and insect herbivores. Annu. Rev. Plant Biol. 70, 527–557. doi: 10.1146/annurev-arplant-050718-095910, PMID: 30786233

[ref30] Falara V. Akhtar T. A. Nguyen T. T. H. Spyropoulou E. A. Bleeker P. M. Schauvinhold I.. (2011). The tomato terpene synthase gene family. Plant Physiol. 157, 770–789. doi: 10.1104/pp.111.179648, PMID: 21813655 PMC3192577

[ref31] Fattorini R. Glover B. J. (2020). Molecular mechanisms of pollination biology. Annu. Rev. Plant Biol. 71, 487–515. doi: 10.1146/annurev-arplant-081519-040003, PMID: 32160004

[ref32] Fenster C. B. Armbruster W. S. Wilson P. Dudash M. R. Thomson J. D. (2004). Pollination syndromes and floral specialization. Annu. Rev. Ecol. Evol. Syst. 35, 375–403. doi: 10.1146/annurev.ecolsys.34.011802.132347

[ref33] Ferrieri A. P. Arce C. C. M. Machado R. A. R. Meza-Canales I. D. Lima E. Baldwin I. T.. (2015). A *Nicotiana attenuata* cell wall invertase inhibitor (NaCWII) reduces growth and increases secondary metabolite biosynthesis in herbivore-attacked plants. New Phytol. 208, 519–530. doi: 10.1111/nph.13475, PMID: 26017581

[ref34] Fowler J. H. Narváez-Vásquez J. Aromdee D. N. Pautot V. Holzer F. M. Walling L. L. (2009). Leucine aminopeptidase regulates defense and wound signaling in tomato downstream of jasmonic acid. Plant Cell 21, 1239–1251. doi: 10.1105/tpc.108.065029, PMID: 19376935 PMC2685619

[ref35] Friedman M. Levin C. E. (1995). α-Tomatine content in tomato and tomato products determined by HPLC with pulsed amperometric detection. J. Agric. Food Chem. 43, 1507–1511. doi: 10.1021/jf00054a017

[ref36] Fürstenberg-Hägg J. Zagrobelny M. Bak S. (2013). Plant defense against insect herbivores. Int. J. Mol. Sci. 14, 10242–10297. doi: 10.3390/ijms140510242, PMID: 23681010 PMC3676838

[ref37] Gatehouse J. A. (2002). Plant resistance towards insect herbivores: a dynamic interaction. New Phytol. 156, 145–169. doi: 10.1046/j.1469-8137.2002.00519.x, PMID: 33873279

[ref38] Giuliano G. Bartley G. E. Scolnik P. A. (1993). Regulation of carotenoid biosynthesis during tomato development. Plant Cell 5, 379–387. doi: 10.1105/tpc.5.4.379, PMID: 8485401 PMC160278

[ref39] González-teuber M. Heil M. (2009). Nectar chemistry is tailored for both attraction of mutualists and protection from exploiters. Plant Signal. Behav. 4, 809–813. doi: 10.4161/psb.4.9.9393, PMID: 19847105 PMC2802787

[ref40] Halitschke R. Gase K. Hui D. Schmidt D. D. Baldwin I. T. (2003). Molecular interactions between the specialist herbivore *Manduca sexta* (Lepidoptera, Sphingidae) and its natural host *Nicotiana attenuata*. VI. Microarray analysis reveals that most herbivore-specific transcriptional changes are mediated by fatty acid-amino acid conjugates. Plant Physiol. 131, 1894–1902. doi: 10.1104/pp.102.018184, PMID: 12692348 PMC166945

[ref41] Halitschke R. Hamilton J. G. Kessler A. (2011). Herbivore-specific elicitation of photosynthesis by mirid bug salivary secretions in the wild tobacco *Nicotiana attenuata*. New Phytol. 191, 528–535. doi: 10.1111/j.1469-8137.2011.03701.x, PMID: 21443673

[ref42] Heil M. (2011). Nectar: generation, regulation and ecological functions. Trends Plant Sci. 16, 191–200. doi: 10.1016/j.tplants.2011.01.003, PMID: 21345715

[ref43] Howe G. A. (2004). Jasmonates as signals in the wound response. J. Plant Growth Regul. 23, 223–237. doi: 10.1007/s00344-004-0030-6

[ref44] Huang X. Z. Chen J. Y. Xiao H. J. Xiao Y. T. Wu J. Wu J. X.. (2015). Dynamic transcriptome analysis and volatile profiling of *Gossypium hirsutum* in response to the cotton bollworm *Helicoverpa armigera*. Sci. Rep. 5:11867. doi: 10.1038/srep11867, PMID: 26148847 PMC4493570

[ref45] Huang H. Liu B. Liu L. Song S. (2017). Jasmonate action in plant growth and development. J. Exp. Bot. 68, 1349–1359. doi: 10.1093/jxb/erw495, PMID: 28158849

[ref46] Huerta-Cepas J. Forslund K. Coelho L. P. Szklarczyk D. Jensen L. J. von Mering C.. (2017). Fast genome-wide functional annotation through orthology assignment by eggNOG-mapper. Mol. Biol. Evol. 34, 2115–2122. doi: 10.1093/molbev/msx148, PMID: 28460117 PMC5850834

[ref47] Huerta-Cepas J. Szklarczyk D. Heller D. Hernández-Plaza A. Forslund S. K. Cook H.. (2019). eggNOG 5.0: a hierarchical, functionally and phylogenetically annotated orthology resource based on 5090 organisms and 2502 viruses. Nucleic Acids Res. 47, D309–D314. doi: 10.1093/nar/gky1085, PMID: 30418610 PMC6324079

[ref48] Iqbal N. Khan N. A. Ferrante A. Trivellini A. Francini A. Khan M. I. R. (2017). Ethylene role in plant growth, development and senescence: interaction with other phytohormones. Front. Plant Sci. 8:475. doi: 10.3389/fpls.2017.00475, PMID: 28421102 PMC5378820

[ref49] Ishiguro S. Kawai-Oda A. Ueda J. Nishida I. Okada K. (2001). The *DEFECTIVE IN ANTHER DEHISCENCE1* gene encodes a novel phospholipase A1 catalyzing the initial step of jasmonic acid biosynthesis, which synchronizes pollen maturation, anther DEHISCENCE, and flower opening in *Arabidopsis*. Plant Cell 13, 2191–2209. doi: 10.1105/tpc.010192, PMID: 11595796 PMC139153

[ref50] Itkin M. Rogachev I. Alkan N. Rosenberg T. Malitsky S. Masini L.. (2011). GLYCOALKALOID METABOLISM1 is required for steroidal alkaloid glycosylation and prevention of phytotoxicity in tomato. Plant Cell 23, 4507–4525. doi: 10.1105/tpc.111.088732, PMID: 22180624 PMC3269880

[ref51] Iwao K. Rausher M. D. (1997). Evolution of plant resistance to multiple herbivores: quantifying diffuse coevolution. Am. Nat. 149, 316–335. doi: 10.1086/285992

[ref52] Jacobsen D. J. Raguso R. A. (2018). Lingering effects of herbivory and plant defenses on pollinators. Curr. Biol. 28, R1164–R1169. doi: 10.1016/j.cub.2018.08.010, PMID: 30300606

[ref53] Kawabata S. Li Y. Saito T. Zhou B. (2009). Identification of differentially expressed genes during flower opening by suppression subtractive hybridization and cDNA microarray analysis in *Eustoma grandiflorum*. Sci. Hortic. 122, 129–133. doi: 10.1016/j.scienta.2009.03.011

[ref54] Kessler A. Halitschke R. Baldwin I. T. (2004). Silencing the jasmonate cascade: induced plant defenses and insect populations. Science 305, 665–668. doi: 10.1126/science.1096931, PMID: 15232071

[ref55] Kim D. Pertea G. Trapnell C. Pimentel H. Kelley R. Salzberg S. L. (2013). TopHat2: accurate alignment of transcriptomes in the presence of insertions, deletions and gene fusions. Genome Biol. 14:R36. doi: 10.1186/gb-2013-14-4-r36, PMID: 23618408 PMC4053844

[ref56] Kolde R. (2019). pheatmap: pretty heatmaps. R package version 1.0.12.

[ref57] Lanahan M. B. Yen H.-C. Giovannoni J. J. Klee H. J. (1994). The *never ripe* mutation blocks ethylene perception in tomato. Plant Cell 6, 521–530. doi: 10.1105/tpc.6.4.521, PMID: 8205003 PMC160455

[ref58] Lee S. Badieyan S. Bevan D. R. Herde M. Gatz C. Tholl D. (2010). Herbivore-induced and floral homoterpene volatiles are biosynthesized by a single P450 enzyme (CYP82G1) in *Arabidopsis*. Proc. Natl. Acad. Sci. U. S. A. 107, 21205–21210. doi: 10.1073/pnas.1009975107, PMID: 21088219 PMC3000306

[ref59] Li B. Dewey C. N. (2011). RSEM: accurate transcript quantification from RNA-seq data with or without a reference genome. BMC Bioinf. 12:323. doi: 10.1186/1471-2105-12-323, PMID: 21816040 PMC3163565

[ref60] Li J. Halitschke R. Li D. Paetz C. Su H. Heiling S.. (2021). Controlled hydroxylations of diterpenoids allow for plant chemical defense without autotoxicity. Science 371, 255–260. doi: 10.1126/science.abe4713, PMID: 33446550

[ref61] Li C. Schilmiller A. L. Liu G. Lee G. I. Jayanty S. Sageman C.. (2005). Role of β-oxidation in jasmonate biosynthesis and systemic wound signaling in tomato. Plant Cell 17, 971–986. doi: 10.1105/tpc.104.029108, PMID: 15722469 PMC1069712

[ref62] Lichtenthaler H. K. (1999). The 1-deoxy-D-xylulose-5-phosphate pathway of isoprenoid biosynthesis in plants. Annu. Rev. Plant Physiol. Plant Mol. Biol. 50, 47–65. doi: 10.1146/annurev.arplant.50.1.47, PMID: 15012203

[ref63] Livak K. J. Schmittgen T. D. (2001). Analysis of relative gene expression data using real-time quantitative PCR and the 2-ΔΔCT method. Methods 25, 402–408. doi: 10.1006/meth.2001.1262, PMID: 11846609

[ref64] Machado R. A. R. Arce C. C. M. Ferrieri A. P. Baldwin I. T. Erb M. (2015). Jasmonate-dependent depletion of soluble sugars compromises plant resistance to *Manduca sexta*. New Phytol. 207, 91–105. doi: 10.1111/nph.13337, PMID: 25704234

[ref65] Machado R. A. R. Baldwin I. T. Erb M. (2017). Herbivory-induced jasmonates constrain plant sugar accumulation and growth by antagonizing gibberellin signaling and not by promoting secondary metabolite production. New Phytol. 215, 803–812. doi: 10.1111/nph.14597, PMID: 28631319

[ref66] Machado R. A. R. Ferrieri A. P. Robert C. A. M. Glauser G. Kallenbach M. Baldwin I. T.. (2013). Leaf-herbivore attack reduces carbon reserves and regrowth from the roots via jasmonate and auxin signaling. New Phytol. 200, 1234–1246. doi: 10.1111/nph.12438, PMID: 23914830

[ref67] McConn M. Creelman R. A. Bell E. Mullet J. E. Browse J. (1997). Jasmonate is essential for insect defense in *Arabidopsis*. Proc. Natl. Acad. Sci. U. S. A. 94, 5473–5477. doi: 10.1073/pnas.94.10.5473, PMID: 11038546 PMC24703

[ref68] Memelink J. (2009). Regulation of gene expression by jasmonate hormones. Phytochemistry 70, 1560–1570. doi: 10.1016/j.phytochem.2009.09.004, PMID: 19796781

[ref69] Milner S. E. Brunton N. P. Jones P. W. O’Brien N. M. Collins S. G. Maguire A. R. (2011). Bioactivities of glycoalkaloids and their aglycones from *Solanum* species. J. Agric. Food Chem. 59, 3454–3484. doi: 10.1021/jf200439q, PMID: 21401040

[ref70] Montero-Vargas J. M. Casarrubias-Castillo K. Martínez-Gallardo N. Ordaz-Ortiz J. J. Délano-Frier J. P. Winkler R. (2018). Modulation of steroidal glycoalkaloid biosynthesis in tomato (*Solanum lycopersicum*) by jasmonic acid. Plant Sci. 277, 155–165. doi: 10.1016/j.plantsci.2018.08.020, PMID: 30466581

[ref71] Muhlemann J. K. Maeda H. Chang C. Miguel P. S. Baxter I. Cooper B.. (2012). Developmental changes in the metabolic network of snapdragon flowers. PLoS One 7:e40381. doi: 10.1371/journal.pone.0040381, PMID: 22808147 PMC3394800

[ref72] Nakayasu M. Shioya N. Shikata M. Thagun C. Abdelkareem A. Okabe Y.. (2018). JRE4 is a master transcriptional regulator of defense-related steroidal glycoalkaloids in tomato. Plant J. 94, 975–990. doi: 10.1111/tpj.13911, PMID: 29569783

[ref73] Niwa T. Suzuki T. Takebayashi Y. Ishiguro R. Higashiyama T. Sakakibara H.. (2018). Jasmonic acid facilitates flower opening and floral organ development through the upregulated expression of *SlMYB21* transcription factor in tomato. Biosci. Biotechnol. Biochem. 82, 292–303. doi: 10.1080/09168451.2017.1422107, PMID: 29448919

[ref74] Pashalidou F. G. Lambert H. Peybernes T. Mescher M. C. De Moraes C. M. (2020). Bumble bees damage plant leaves and accelerate flower production when pollen is scarce. Science 368, 881–884. doi: 10.1126/science.aay0496, PMID: 32439792

[ref75] Paudel S. Lin P. A. Foolad M. R. Ali J. G. Rajotte E. G. Felton G. W. (2019). Induced plant defenses against herbivory in cultivated and wild tomato. J. Chem. Ecol. 45, 693–707. doi: 10.1007/s10886-019-01090-4, PMID: 31367970

[ref76] Pease J. B. Haak D. C. Hahn M. W. Moyle L. C. (2016). Phylogenomics reveals three sources of adaptive variation during a rapid radiation. PLoS Biol. 14:e1002379. doi: 10.1371/journal.pbio.1002379, PMID: 26871574 PMC4752443

[ref77] Peng S. Huang S. Liu Z. Feng H. (2019). Mutation of *ACX1*, a jasmonic acid biosynthetic enzyme, leads to petal degeneration in Chinese cabbage (*Brassica campestris* ssp. *pekinensis*). Int. J. Mol. Sci. 20:2310. doi: 10.3390/ijms20092310, PMID: 31083282 PMC6539522

[ref78] Pertea M. Pertea G. M. Antonescu C. M. Chang T.-C. Mendell J. T. Salzberg S. L. (2015). StringTie enables improved reconstruction of a transcriptome from RNA-seq reads. Nat. Biotechnol. 33, 290–295. doi: 10.1038/nbt.3122, PMID: 25690850 PMC4643835

[ref79] Qi J. Malook S. Shen G. Gao L. Zhang C. Li J.. (2018). Current understanding of maize and rice defense against insect herbivores. Plant Divers. 40, 189–195. doi: 10.1016/j.pld.2018.06.006, PMID: 30740564 PMC6137261

[ref80] Ralph S. G. Yueh H. Friedmann M. Aeschliman D. Zeznik J. A. Nelson C. C.. (2006). Conifer defence against insects: microarray gene expression profiling of Sitka spruce (*Picea sitchensis*) induced by mechanical wounding or feeding by spruce budworms (*Choristoneura occidentalis*) or white pine weevils (*Pissodes strobi*) reveals large-scale changes of the host transcriptome. Plant Cell Environ. 29, 1545–1570. doi: 10.1111/j.1365-3040.2006.01532.x, PMID: 16898017

[ref81] Ramos S. E. Schiestl F. P. (2019). Rapid plant evolution driven by the interaction of pollination and herbivory. Science 364, 193–196. doi: 10.1126/science.aav6962, PMID: 30975889

[ref82] Ramos S. E. Schiestl F. P. (2020). Evolution of floral fragrance is compromised by herbivory. Front. Ecol. Evol. 8:30. doi: 10.3389/fevo.2020.00030

[ref83] Rasmann S. Vilas J. S. Glauser G. Cartolano M. Lempe J. Tsiantis M.. (2018). Pleiotropic effect of the *flowering locus C* on plant resistance and defence against insect herbivores. J. Ecol. 106, 1244–1255. doi: 10.1111/1365-2745.12894

[ref84] Robinson M. D. McCarthy D. J. Smyth G. K. (2010). edgeR: a bioconductor package for differential expression analysis of digital gene expression data. Bioinformatics 26, 139–140. doi: 10.1093/bioinformatics/btp616, PMID: 19910308 PMC2796818

[ref85] Roddick J. G. (1996). Steroidal glycoalkaloids: nature and consequences of bioactivity. Adv. Exp. Med. Biol. 404, 277–295. doi: 10.1007/978-1-4899-1367-8_25, PMID: 8957303

[ref86] Rodríguez V. M. Santiago R. Malvar R. A. Butrón A. (2012). Inducible maize defense mechanisms against the corn borer *Sesamia nonagrioides*: a transcriptome and biochemical approach. Mol. Plant-Microbe Interact. 25, 61–68. doi: 10.1094/MPMI-06-11-0154, PMID: 21916555

[ref87] Rogachev I. Aharoni A. (2012). UPLC-MS-based metabolite analysis in tomato. Methods in Mol. Biol. 860, 129–144. doi: 10.1007/978-1-61779-594-7_9 22351175

[ref88] Rohmer M. (1999). The discovery of a mevalonate-independent pathway for isoprenoid biosynthesis in bacteria, algae and higher plants. Nat. Prod. Rep. 16, 565–574. doi: 10.1039/a709175c, PMID: 10584331

[ref89] Rusman Q. Poelman E. H. Nowrin F. Polder G. Lucas-Barbosa D. (2019). Floral plasticity: herbivore-species-specific-induced changes in flower traits with contrasting effects on pollinator visitation. Plant Cell Environ. 42, 1882–1896. doi: 10.1111/pce.13520, PMID: 30659631 PMC6850075

[ref90] Schnee C. Köllner T. G. Held M. Turlings T. C. J. Gershenzon J. Degenhardt J. (2006). The products of a single maize sesquiterpene synthase form a volatile defense signal that attracts natural enemies of maize herbivores. Proc. Natl. Acad. Sci. U. S. A. 103, 1129–1134. doi: 10.1073/pnas.0508027103, PMID: 16418295 PMC1347987

[ref91] Schwacke R. Ponce-Soto G. Y. Krause K. Bolger A. M. Arsova B. Hallab A.. (2019). MapMan4: a refined protein classification and annotation framework applicable to multi-omics data analysis. Mol. Plant 12, 879–892. doi: 10.1016/j.molp.2019.01.003, PMID: 30639314

[ref92] Shalit-Kaneh A. Eviatar-Ribak T. Horev G. Suss N. Aloni R. Eshed Y.. (2019). The flowering hormone florigen accelerates secondary cell wall biogenesis to harmonize vascular maturation with reproductive development. Proc. Natl. Acad. Sci. U. S. A. 116, 16127–16136. doi: 10.1073/pnas.1906405116, PMID: 31324744 PMC6690031

[ref93] Sheehan H. Hermann K. Kuhlemeier C. (2012). Color and scent: how single genes influence pollinator attraction. Cold Spring Harb. Symp. Quant. Biol. 77, 117–133. doi: 10.1101/sqb.2013.77.014712, PMID: 23467550

[ref94] Shinozaki Y. Hao S. Kojima M. Sakakibara H. Ozeki-iida Y. Zheng Y.. (2015). Ethylene suppresses tomato (*Solanum lycopersicum*) fruit set through modification of gibberellin metabolism. Plant J. 83, 237–251. doi: 10.1111/tpj.12882, PMID: 25996898

[ref95] Simms E. L. Fritz R. S. (1990). The ecology and evolution of host-plant resistance to insects. Trends Ecol. Evol. 5, 356–360. doi: 10.1016/0169-5347(90)90094-T, PMID: 21232392

[ref96] Stitz M. Hartl M. Baldwin I. T. Gaquerel E. (2014). Jasmonoyl-L-isoleucine coordinates metabolic networks required for anthesis and floral attractant emission in wild tobacco (*Nicotiana attenuata*). Plant Cell 26, 3964–3983. doi: 10.1105/tpc.114.128165, PMID: 25326292 PMC4247565

[ref97] Strauss S. Y. Irwin R. E. (2004). Ecological and evolutionary consequences of multispecies plant-animal interactions. Annu. Rev. Ecol. Evol. Syst. 35, 435–466. doi: 10.1146/annurev.ecolsys.35.112202.130215

[ref98] Thimm O. Bläsing O. Gibon Y. Nagel A. Meyer S. Krüger P.. (2004). MAPMAN: a user-driven tool to display genomics data sets onto diagrams of metabolic pathways and other biological processes. Plant J. 37, 914–939. doi: 10.1111/j.1365-313X.2004.02016.x, PMID: 14996223

[ref99] Tieman D. M. Taylor M. G. Ciardi J. A. Klee H. J. (2000). The tomato ethylene receptors NR and LeETR4 are negative regulators of ethylene response and exhibit functional compensation within a multigene family. Proc. Natl. Acad. Sci. U. S. A. 97, 5663–5668. doi: 10.1073/pnas.090550597, PMID: 10792050 PMC25885

[ref100] Umemoto N. Nakayasu M. Ohyama K. Yotsu-Yamashita M. Mizutani M. Seki H.. (2016). Two cytochrome P450 monooxygenases catalyze early hydroxylation steps in the potato steroid glycoalkaloid biosynthetic pathway. Plant Physiol. 171, 2458–2467. doi: 10.1104/pp.16.00137, PMID: 27307258 PMC4972264

[ref101] Vergauwen R. Van den Ende W. Van Laere A. (2000). The role of fructan in flowering of *Campanula rapunculoides*. J. Exp. Bot. 51, 1261–1266. doi: 10.1093/jexbot/51.348.1261, PMID: 10937702

[ref102] Voelckel C. Baldwin I. T. (2004). Generalist and specialist lepidopteran larvae elicit different transcriptional responses in *Nicotiana attenuata*, which correlate with larval FAC profiles. Ecol. Lett. 7, 770–775. doi: 10.1111/j.1461-0248.2004.00633.x

[ref103] Voelckel C. Weisser W. W. Baldwin I. T. (2004). An analysis of plant-aphid interactions by different microarray hybridization strategies. Mol. Ecol. 13, 3187–3195. doi: 10.1111/j.1365-294X.2004.02297.x, PMID: 15367131

[ref104] Walling L. L. (2000). The myriad plant responses to herbivores. J. Plant Growth Regul. 19, 195–216. doi: 10.1007/s003440000026, PMID: 11038228

[ref105] Wang L. Wu J. (2013). The essential role of jasmonic acid in plant-herbivore interactions -using the wild tobacco *Nicotiana attenuata* as a model. J. Genet. Genomics 40, 597–606. doi: 10.1016/j.jgg.2013.10.001, PMID: 24377866

[ref106] Wang J. Wu D. Wang Y. Xie D. (2019). Jasmonate action in plant defense against insects. J. Exp. Bot. 70, 3391–3400. doi: 10.1093/jxb/erz174, PMID: 30976791

[ref107] War A. R. Paulraj M. G. Ahmad T. Buhroo A. A. Hussain B. Ignacimuthu S.. (2012). Mechanisms of plant defense against insect herbivores. Plant Signal. Behav. 7, 1306–1320. doi: 10.4161/psb.21663, PMID: 22895106 PMC3493419

[ref108] Wasternack C. Forner S. Strnad M. Hause B. (2013). Jasmonates in flower and seed development. Biochimie 95, 79–85. doi: 10.1016/j.biochi.2012.06.005, PMID: 22705387

[ref109] Wasternack C. Hause B. (2013). Jasmonates: biosynthesis, perception, signal transduction and action in plant stress response, growth and development. An update to the 2007 review in annals of botany. Ann. Bot. 111, 1021–1058. doi: 10.1093/aob/mct067, PMID: 23558912 PMC3662512

[ref110] Wise M. J. Rausher M. D. (2013). Evolution of resistance to a multiple-herbivore community: genetic correlations, diffuse coevolution, and constraints on the plant’s response to selection. Evolution 67, 1767–1779. doi: 10.1111/evo.12061, PMID: 23730768

[ref111] Wu J. Baldwin I. T. (2010). New insights into plant responses to the attack from insect herbivores. Annu. Rev. Genet. 44, 1–24. doi: 10.1146/annurev-genet-102209-163500, PMID: 20649414

[ref112] Xiao Y. Chen Y. Charnikhova T. Mulder P. P. J. Heijmans J. Hoogenboom A.. (2014). *OsJAR1* is required for JA-regulated floret opening and anther dehiscence in rice. Plant Mol. Biol. 86, 19–33. doi: 10.1007/s11103-014-0212-y, PMID: 24947835

[ref113] Xu S. Kreitzer C. McGale E. Lackus N. D. Guo H. Köllner T. G.. (2020). Allelic differences of clustered terpene synthases contribute to correlated intraspecific variation of floral and herbivory-induced volatiles in a wild tobacco. New Phytol. 228, 1083–1096. doi: 10.1111/nph.16739, PMID: 32535930

[ref114] Yan L. Zhai Q. Wei J. Li S. Wang B. Huang T.. (2013). Role of tomato lipoxygenase D in wound-induced jasmonate biosynthesis and plant immunity to insect herbivores. PLoS Genet. 9:e1003964. doi: 10.1371/journal.pgen.1003964, PMID: 24348260 PMC3861047

[ref115] Yap Y. M. Loh C. S. Ong B. L. (2008). Regulation of flower development in *Dendrobium crumenatum* by changes in carbohydrate contents, water status and cell wall metabolism. Sci. Hortic. 119, 59–66. doi: 10.1016/j.scienta.2008.06.029

[ref116] Yu G. Wang L. G. Han Y. He Q. Y. (2012). clusterProfiler: an R package for comparing biological themes among gene clusters. OMICS 16, 284–287. doi: 10.1089/omi.2011.0118, PMID: 22455463 PMC3339379

[ref117] Yuan Z. Zhang D. (2015). Roles of jasmonate signalling in plant inflorescence and flower development. Curr. Opin. Plant Biol. 27, 44–51. doi: 10.1016/j.pbi.2015.05.024, PMID: 26125498

[ref118] Zhang Y. Bouwmeester H. J. Kappers I. F. (2020). Combined transcriptome and metabolome analysis identifies defence responses in spider mite-infested pepper (*Capsicum annuum*). J. Exp. Bot. 71, 330–343. doi: 10.1093/jxb/erz422, PMID: 31557301 PMC6913709

[ref119] Zhang L. Zhang F. Melotto M. Yao J. He S. Y. (2017). Jasmonate signaling and manipulation by pathogens and insects. J. Exp. Bot. 68, 1371–1385. doi: 10.1093/jxb/erw478, PMID: 28069779 PMC6075518

[ref120] Zhou W. Kügler A. McGale E. Haverkamp A. Knaden M. Guo H.. (2017). Tissue-specific emission of (*E*)-α-bergamotene helps resolve the dilemma when pollinators are also herbivores. Curr. Biol. 27, 1336–1341. doi: 10.1016/j.cub.2017.03.017, PMID: 28434859

[ref121] Zhou F. Pichersky E. (2020). The complete functional characterisation of the terpene synthase family in tomato. New Phytol. 226, 1341–1360. doi: 10.1111/nph.16431, PMID: 31943222 PMC7422722

[ref122] Zhu-salzman K. Salzman R. A. Ahn J. Koiwa H. (2004). Transcriptional regulation of sorghum defense determinants against a phloem-feeding aphid. Plant Physiol. 134, 420–431. doi: 10.1104/pp.103.028324, PMID: 14701914 PMC316321

[ref123] Zu P. Blanckenhorn W. U. Schiestl F. P. (2016). Heritability of floral volatiles and pleiotropic responses to artificial selection in *Brassica rapa*. New Phytol. 209, 1208–1219. doi: 10.1111/nph.13652, PMID: 26391626

