# Supplementary material for: Transcriptomic Profiling Reveals Shared Signalling Networks Between Flower Development and Herbivory-Induced Responses in Tomato
Source: Front Plant Sci. 2021 Sep 21;12:722810. doi: 10.3389/fpls.2021.722810 (PMC8493932; doi:10.3389/fpls.2021.722810)
Supplement: Supplementary file 2 [file Data_Sheet_2.docx]

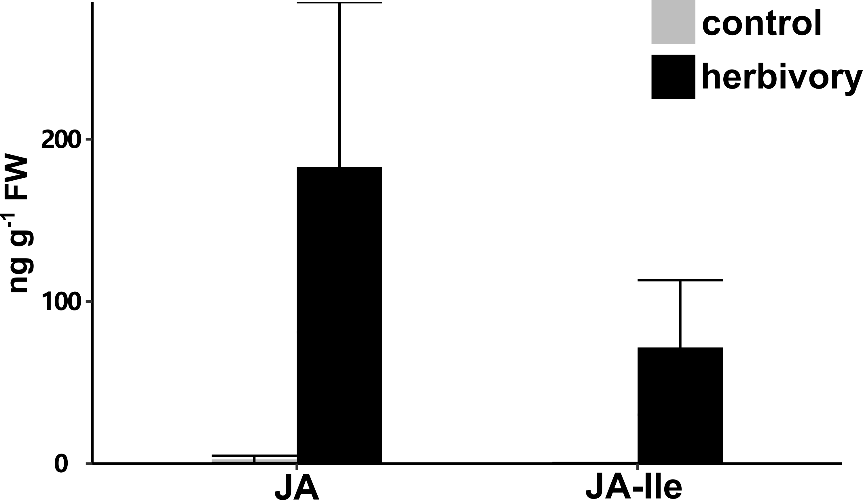


**Supplementary Figure 1. Herbivory-induced accumulation of JA and JA-Ile.** Average abundance (+ standard error; n=3) of JA and JA-Ile in control (grey bar) and herbivore-infested (black bar) leaves of domesticated tomato. FW, fresh weight. Due to low sample size and low statistical power, the differences are not statistically different (*P* value for JA and JA-Ile is 0.15 and 0.16, respectively, one-way ANOVA).


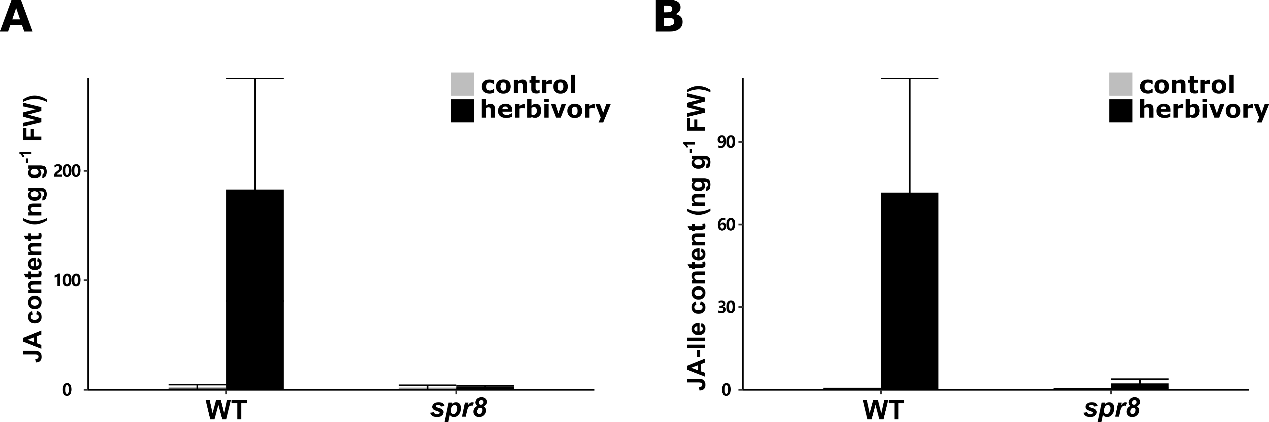


**Supplementary Figure 2. Herbivory-induced accumulation of JA and JA-Ile is suppressed in *spr8* plants.** Average abundance (+ standard error; n=3) of JA **(A)** and JA-Ile **(B)** in control (grey bar) and herbivore-infested (black bar) leaves of WT and *spr8* plants*.* FW, fresh weight. Due to low sample size, the differences between treatments and genotypes are not significant (for JA, *P* values for genotype and treatment are 0.11, for JA-Ile, P values for genotype and treatment are 0.13 and 0.11, respectively).


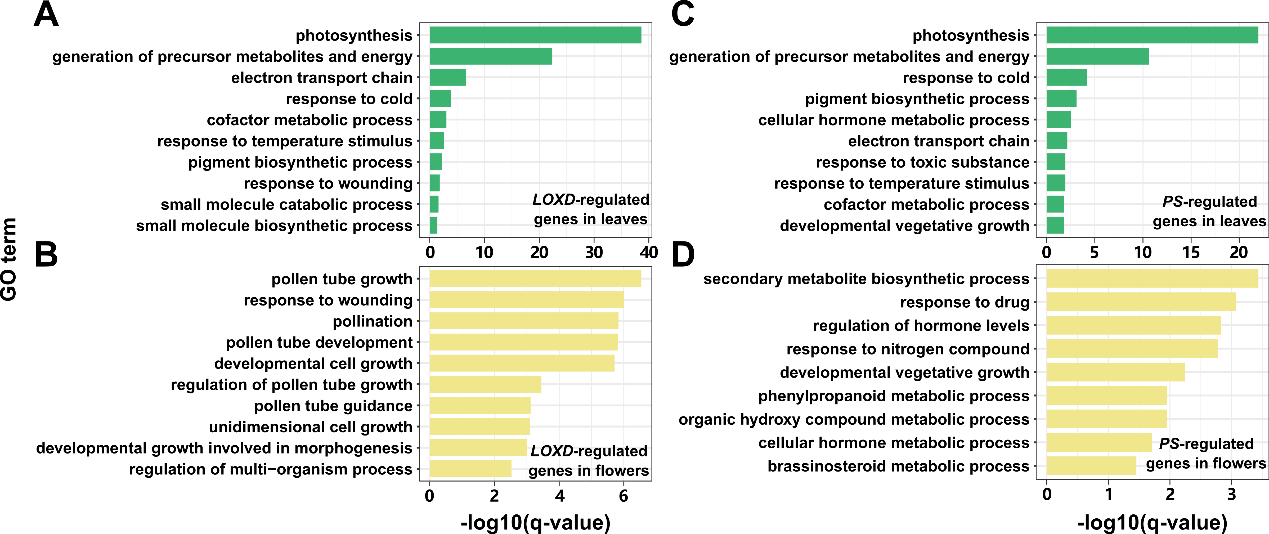


**Supplementary Figure 3. GO enrichment analysis for *LOXD*/*PS*-regulated genes in leaves and flowers of tomato.** *LOXD*-regulated genes (DEGs between WT and *spr8* plants) in **(A)** herbivore-infested leaves and **(B)** during flower development (stage 4). *PS*-regulated genes (DEGs between WT and *35S::PS* plants) in **(C)** untreated control leaves and **(D)** during flower development (stage 5).


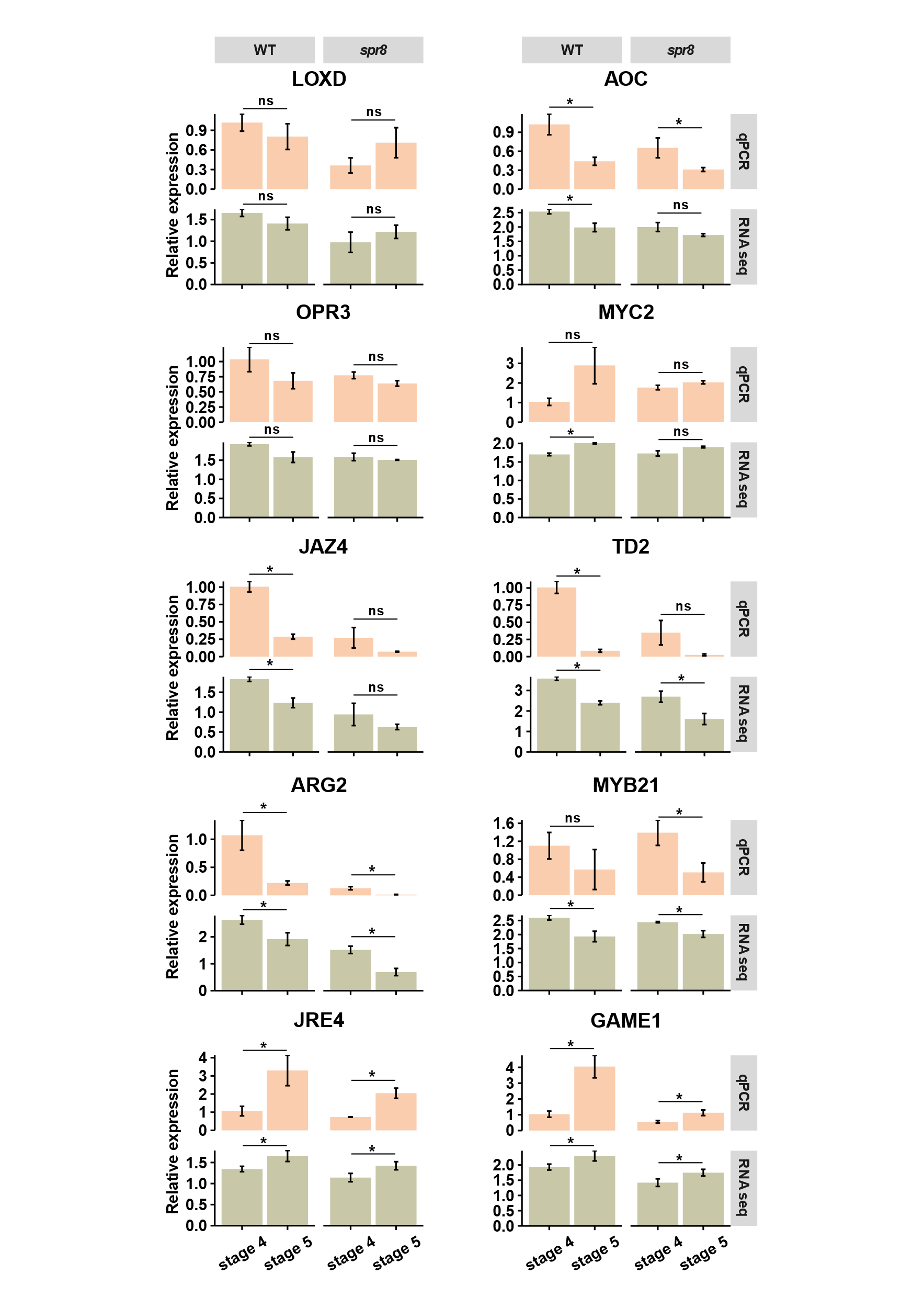


**Supplementary Figure 4.** **qPCR validation of 10 selected genes between two flower stages of two tomato genotypes.** Relative expression values for qPCR were calculated using reference gene *UBI3* by 2^-ΔΔCt^ method. Log10(TPM+1) were represented as relative expression levels for RNA-seq. * indicates the expression level of the certain gene between two stages are significantly different (for qPCR, *P* value ≤ 0.05, t-test; for RNA-seq, FDR ≤ 0.05); *ns*, not significant.


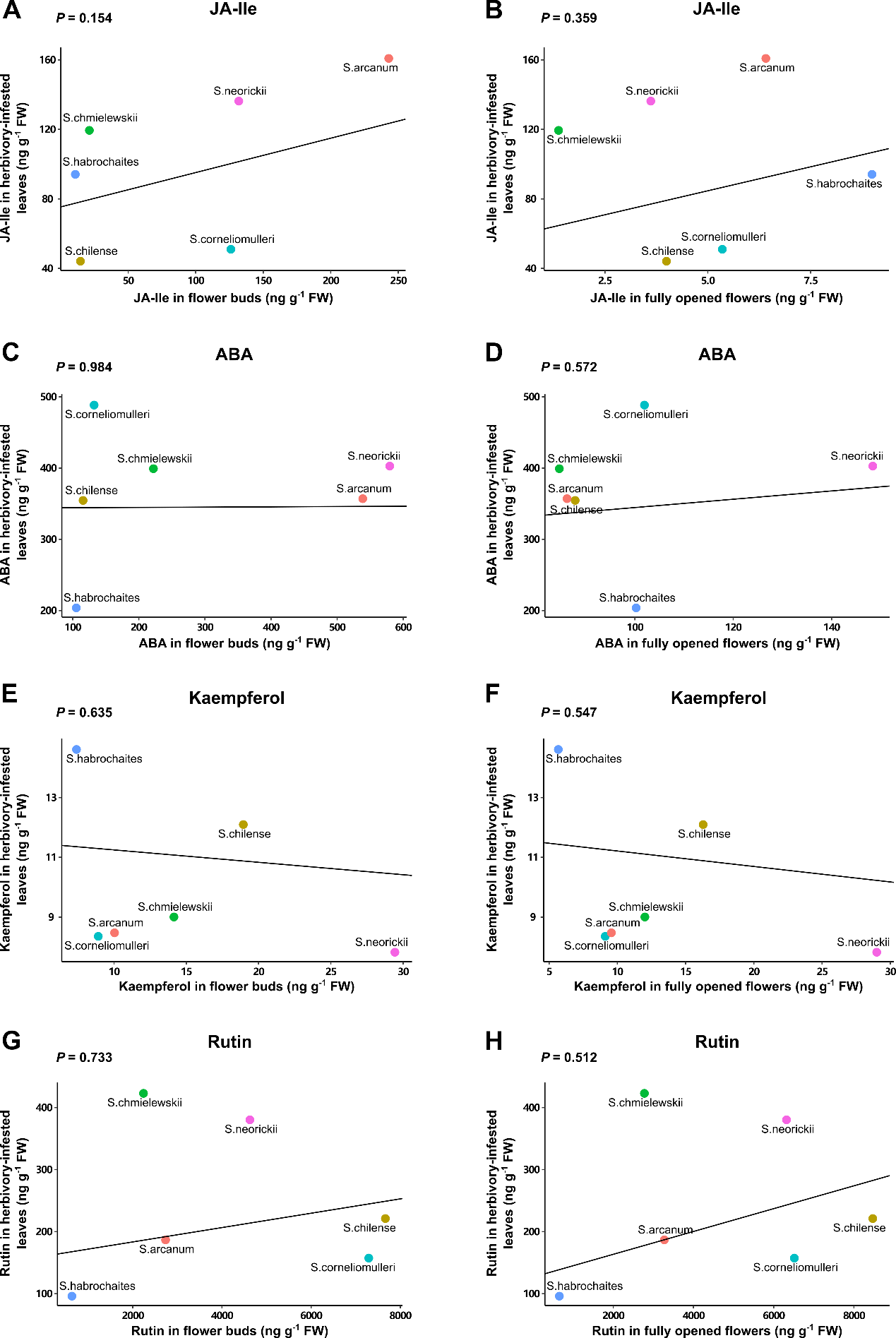
**Supplementary Figure 5. Correlations of other specialized metabolites and phytohormones in tomato leaves and flowers. (A, B)** Correlations JA-Ile between herbivory-infested leaves and flower buds **(A)** or fully opened flowers **(B)** among six wild tomato species. **(C, D)** Correlations of ABA between herbivory-infested leaves and flower buds **(C)** or fully opened flowers **(D)** among six wild tomato species. **(E, F)** Correlations of kaempferol between herbivory-infested leaves and flower buds **(E)** or fully opened flowers **(F)** among six wild tomato species. **(G, H)** Correlations of rutin between herbivory-infested leaves and flower buds **(G)** or fully opened flowers **(H)** among six wild tomato species. Considering the phylogenetic relationship, correlations were analysed using a generalized least squares method (GLS) with a phylogenetic tree based on the phylogeny of *Solanum* sect. *Lycopersicon* from Pease *et al.* (2016).

**REFERENCES**

Pease, J. B., Haak, D. C., Hahn, M. W., and Moyle, L. C. (2016). Phylogenomics reveals three sources of adaptive variation during a rapid radiation. *PLoS Biol.* 14, e1002379. doi:10.1371/journal.pbio.1002379.
